# Health expenditures by services and providers for 195 countries, 2000–2017

**DOI:** 10.1136/bmjgh-2021-005799

**Published:** 2021-07-30

**Authors:** Matthew T Schneider, Angela Y Chang, Abigail Chapin, Catherine S Chen, Sawyer W Crosby, Anton C Harle, Golsum Tsakalos, Bianca S Zlavog, Joseph L Dieleman

**Affiliations:** 1 Institute for Health Metrics and Evaluation, Seattle, Washington, USA; 2 Institute for Disease Modeling, Seattle, Washington, USA; 3 Danish Institute for Advanced Study, Copenhagen, Denmark; 4 Department of Clinical Research, University of Southern Denmark, Odense, Denmark

**Keywords:** health economics

## Abstract

**Introduction:**

National Health Accounts are a significant source of health expenditure data, designed to be comprehensive and comparable across countries. However, there is currently no single repository of this data and even when compiled major gaps persist. This research aims to provide policymakers and researchers with a single repository of available national health expenditures by healthcare functions (ie, services) and providers of such services. Leveraging these data within statistical methods, a complete set of detailed health expenditures is estimated.

**Methods:**

A methodical compilation and synthesis of all available national health expenditure reports including disaggregation by healthcare functions and providers was conducted. Using these data, a Bayesian multivariate regression analysis was implemented to estimate national-level health expenditures by the cross-classification of functions and providers for 195 countries, from 2000 to 2017.

**Results:**

This research used 1662 country-years and 110 070 data points of health expenditures from existing National Health Accounts. The most detailed country-year had 52% of the categories of interest reported. Of all health functions, curative care and medical goods were estimated to make up 51.4% (uncertainty interval (UI) 33.2% to 59.4%) and 17.5% (UI 13.0% to 26.9%) of total global health expenditures in 2017, respectively. Three-quarters of the global health expenditures are allocated to three categories of providers: hospital providers (35.4%, UI 30.3% to 38.9%), providers of ambulatory care (25.5%, UI 21.1% to 28.8%) and retailers of medical goods (14.4%, UI 12.4% to 16.3%). As gross domestic product increases, countries spend more on long-term care and less on preventive care.

**Conclusion:**

Disaggregated estimates of health expenditures are often unavailable and unable to provide policymakers and researchers a holistic understanding of how expenditures are used. This research aggregates reported data and provides a complete time-series of estimates, with uncertainty, of health expenditures by health functions and providers between 2000 and 2017 for 195 countries.

**Key questions**

**What is already known?**

- To reach national and global targets, such as the health-related Sustainable Development Goals and Universal Health Coverage, policymakers and researchers need a complete understanding of health expenditures disaggregated by the types of services and who is providing these services.
- To date, National Health Accounts are the single most comprehensive and comparable resource for this information; however, they are often missing details and there is no one single repository of these detailed health expenditures in a usable format.

**What are the new findings?**

- Through methodically searching for and compiling all available National Health Accounts with any amount of health expenditures reported by types of services and providers, 1662 country-years were identified.
- Of 195 countries, 55 reported no such health expenditures between 1995 and 2017, while 28 countries (all but one being high income) reported at least some detailed health expenditures.
- Leveraging these newly compiled data, a statistical analysis was developed to estimate, with uncertainty, all 195 countries’ health expenditures between 2000 and 2017.
- The estimates reflect country-reported data and show that curative care in hospital settings made up the largest share of health expenditures across all income groups, while low-income countries spent a larger share on preventive care than any other income group.

**Key questions**

**What do the new findings imply?**

- Policymakers and researchers now can use the output of this research to review and leverage all available country-reported health expenditures by types of services and providers, as well as, our complete set of estimates of these expenditures for all countries between 2000 and 2017.
- Leveraging these data will allow national and international stakeholders to understand past trends in health expenditures and be used as one piece of information to help inform potential adjustments and course corrections to best reach national and international health targets.

## Introduction

Global health expenditures have risen dramatically in the past two decades, from US$4.1 trillion (8.3% of gross domestic product (GDP)) in 2000 to US$8.2 trillion (9.7% of GDP) in 2017.^1^ Understanding how these health expenditures are spent is essential for policymakers, healthcare providers and payers to be able to maximise healthcare service provision and allocate personnel and other limited resources.^2–8^ National reported health expenditures, particularly from National Health Accounts, are intended to be a key resource for policymakers and researchers to understand differences within and between countries over time.^4 7–12^ Based on the System of Health Accounts (SHA) framework, countries reporting health expenditure using this system focus on disaggregating health expenditures into three pillars: the types of goods and services that are delivered (healthcare functions (HC)), which healthcare providers delivered these goods and services (healthcare providers (HP)), and what financing schemes paid for these goods and services (financing schemes (HF)).^4 9^

Using health expenditures broken down into HC, HP, and HF, researchers and policymakers have set overarching national and international health financing goals, such as reaching Universal Health Coverage and understanding expenditures for primary healthcare.^9 13–15^ However, there is no single repository to easily access this information. Furthermore, available data are inconsistently reported, largely incomplete, and challenging to combine due to variability in formatting, particularly in less developed countries and older reports.^11^ These barriers have inhibited the ability to draw a complete understanding of trends in health financing systems.^6 8 11 16 17^

There are currently two main repositories for country-reported health expenditure data and documents. The first is the Organization for Economic Co-operation and Development (OECD) which collects and makes available health accounts reported by member states and a select number of other countries, including Brazil, China, India and South Africa.^18^ The second repository is the WHO’s Global Health Expenditure Database.^19^ The WHO’s Global Health Expenditure Database collects health account reports from member states and makes them available for download. While these repositories report all three disaggregates of health expenditures (HC, HP and HF), there has been a large amount of work estimating sources of financing and little on HC and HP.^17 20 21^

Within the OECD database, the majority of consistently reported health expenditure data by HC and HP categories are only from high-income countries. WHO’s Global Health Expenditure Database has expenditures mostly from low-income and middle-income countries, however, it has only extracted and made available HC spending since 2016, leaving the majority of low-income and middle- income countries’ data still housed in the original PDF format. Drawing on the WHO and OECD databases, Bui *et al* assessed the completeness of 872 National Health Accounts from the time period of 1996–2010. They found that out of 193 United Nations’ member states, 76 reported no accounts and even where health accounts were reported, they had incomplete reporting of spending by HC and HP categories.^11^

Our research builds on the work by the WHO, OECD, and Bui *et al*, compiling, estimating, and assessing expenditures across HC and HP from 2000 to 2017. We focused on expenditures disaggregated into HC and HP categories because understanding these is essential for health policymakers and existing research has generally focused on understanding financing schemes. To fill the gap in missing data, we estimated a complete time-series of health expenditures for all countries by the cross-tabulation of HC and HP categories.

## Methods

### Search for health expenditure data

A four-step approach was taken to identify all available health accounts. First, National Health Accounts previously collected for the work by Bui *et al* were reviewed and categorised.^11^ Second, the OECD and WHO website repositories were searched for any and all available data or reports.^18 19^ Third, country government websites were reviewed for any additional health account reports not identified or collected in the first two steps. Fourth, keyword searches on the worldwide web were used to identify any additional account reports or data not previously identified. This search was first conducted in 2019 and updated in February 2020. The flow of health account collection is presented in the online supplemental appendix 1. All collected accounts and their sources were uploaded to the Global Health Data Exchange at healthdata.org.

10.1136/bmjgh-2021-005799.supp1 Supplementary data

### Extraction process

All health accounts identified in the search were reviewed for health expenditures reported in categories of HC, HP or the cross-tabulation of HC and HP as defined by the SHA. Three approaches were taken to extract these identified tables: (1) download in current format, (2) automatically convert to Excel and (3) extract by hand. If the same country-year of reported health expenditures were identified from two different reports, the most recently reported data were taken unless issues of completeness or accuracy were identified.

Where the extracted data category names did not match the SHA guidance, two research team members independently reviewed the health account to categorise these expenditures into the most appropriate SHA category. If no category was deemed appropriate, these expenditures were placed into a *not elsewhere classified* (NEC) category. A third researcher reviewed the other team members’ categorisation. If the categories did not align, the third researcher rereviewed the health account to decide which category was most appropriate and consistent.

### Reconciling versions of HC and HP categories

Health expenditures within health accounts were reported by countries following either the SHA 2000 or 2011 guidelines for categorising these data into functions and providers. Between the two guidelines, many categories remain the same, however, some did change. To ensure consistency between the collected raw health expenditure, all estimates originally reported using SHA 2000 guidelines were converted to the updated SHA 2011 framework. SHA 2011 guidelines were used to map between SHA versions.^4^ For certain HC, a category in the SHA 2000 framework was split into multiple SHA 2011 categories but the exact proportioning was undefined. One example is SHA 2011 Immunisation programmes (HC 6.2) which was part of SHA 2000 category Prevention of communicable diseases (HC 6.3). Without knowing how this breakdown should be implemented, these were treated as missing values and allowed to be estimated using the statistical methods. For the complete table of mapping between SHA versions, see the online supplemental appendix table A.2.

All capital formation identified in country reporting from health accounts using SHA 2000 was removed from total health expenditures to align with the SHA 2011 framework. Importantly, if a specific value of HC, HP or any combination of the two categories was not reported by a country, this analysis assumed it was missing, not zero. Data points collected that were reported as zeroes were removed due to their influence on the estimation methods causing systematic underestimation. All expenditures were converted from reported currencies to 2017 US dollars (USD).

### Estimation of complete time-series

After extracting, cleaning, compiling, and reconciling differences of all identified health expenditures reported by HC and HP categories, missing cross-classifications of these data and countries with no accounts were estimated using a Bayesian multivariate model.^22^ The dependent variable was the reported health expenditure for each cross-tabulation of HC and HP as a share of total health expenditure for that given country-year. The dependent variable was then logit transformed to remain bound between 0 and 1. The independent variables were chosen based on data availability, literature review and statistical significance found through a stepwise analysis.^23^ The exact covariates and their sources are discussed below. The Bayesian multivariate model outperformed other multivariate predictive tools based on in/out-of-sample predictive validations. These comparisons can be found in the online supplemental appendix 1.

The estimation method could not constrain the estimates of the HC and HP categories to sum to their respective aggregates. Thus, modelled estimates were ‘raked’ to each component’s higher level. Table 1 lists all HC and HP categories used in this analysis and their respective level label. An example of the level hierarchy would be that curative care (HC 1, level 1) is broken down into inpatient (HC 1.1, level 2), day (HC 1.2, level 2), outpatient (HC 1.3, level 2), home-based (HC 1.4, level 2) and NEC (HC 1.nec, level 2) curative care. This process is followed down to the lowest levels of the cross-classification of HC and HP categories and uses an iterative proportional fitting method to maintain the original proportions from the models while the totals sum correctly in this multidimensional space.^24^ Uncertainty intervals (UIs) for the raked estimates were calculated using the 2.5th and 97.5th percentile of 1000 raked samples for each country-year of the data.

**Table 1:** Estimated global expenditures by healthcare functions and healthcare provider categories

Healthcare functions	Hierarchy level	Estimated global expenditures per capita 2017 US$, (uncertainty intervals)
1. Curative care	1	523 (338 to 604)
1.1 Curative care—inpatient	2	223 (155 to 276)
1.2 Curative care—day	2	20 (10 to 36)
1.3 Curative care—outpatient	2	232 (137 to 291)
1.4 Curative care—home-based	2	6 (4 to 12)
1.nec Curative care—NEC	2	42 (14 to 92)
2. Rehabilitative care	1	25 (12 to 74)
2.1 Rehabilitative care—inpatient	2	11 (5 to 31)
2.2 Rehabilitative care—day	2	2 (1 to 5)
2.3 Rehabilitative care—outpatient	2	6 (2 to 20)
2.4 Rehabilitative care—home-based	2	1 (0 to 5)
2.nec Rehabilitative care—NEC	2	5 (1 to 19)
3. Long-term care	1	76 (55 to 117)
3.1 Long-term care—inpatient	2	49 (29 to 77)
3.2 Long-term care—day	2	4 (2 to 7)
3.3 Long-term care—outpatient	2	1 (0 to 3)
3.4 Long-term care—home-based	2	14 (8 to 23)
3.nec Long-term care—NEC	2	8 (1 to 26)
4. Ancillary care	1	59 (23 to 149)
5. Medical goods	1	178 (132 to 274)
5.1 Medical goods—pharms and other medical non-durable goods	2	136 (92 to 214)
5.2 Medical goods—therapeutic appliances and prosthetics	2	24 (15 to 42)
5.nec Medical goods—NEC	2	18 (5 to 47)
6. Preventative care	1	47 (33 to 72)
6.1 Prevention care—IEC programmes	2	5 (3 to 9)
6.2 Prevention care—immunisation programmes	2	4 (2 to 8)
6.3 Prevention care—early disease detection	2	2 (1 to 4)
6.4 Prevention care—healthy condition monitoring programmes	2	7 (4 to 14)
6.5 Prevention care—EPI surveillance and risk and disease control programmes	2	4 (3 to 7)
6.6 Prevention care—preparing for disaster and emergency response programmes	2	1 (0 to 2)
6.nec Prevention care—NEC	2	23 (14 to 37)
7. Governance and admin	1	64 (40 to 110)
9. Other healthcare services	1	47 (25 to 83)

Healthcare providers	Hierarchy levels	Estimated global expenditures per capita 2017 US$, (uncertainty intervals)
1. Hospitals	1	360 (308 to 396)
1.1 Hospitals—general	2	243 (179 to 303)
1.2 Hospitals—mental health	2	15 (9 to 22)
1.3 Hospitals—specialised	2	32 (20 to 51)
1.nec Hospitals—other	2	70 (34 to 122)
2. Residential long-term care facilities	1	53 (43 to 65)
3. Providers of ambulatory care	1	260 (214 to 293)
3.1 Providers of ambulatory care—medical practices	2	82 (52 to 114)
3.2 Providers of ambulatory care—dental practices	2	33 (19 to 49)
3.3 Providers of ambulatory care—other practices	2	16 (10 to 26)
3.4 Providers of ambulatory care—ambulatory healthcare centres	2	62 (39 to 89)
3.5 Providers of home healthcare services	2	13 (6 to 32)
3.nec Providers of ambulatory care—NEC	2	54 (18 to 112)
4. Providers of ancillary services	1	42 (12 to 136)
5. Retailers and other providers of medical goods	1	147 (126 to 166)
6. Providers of preventative care	1	32 (25 to 39)
7. Healthcare system admin	1	52 (40 to 63)
8. Rest of economy	1	12 (8 to 20)
9. Rest of world	1	18 (4 to 66)
NEC	1	41 (26 to 64)

EPI, Expanded Programme on Immunisation; IEC, Information, Education and Communication; NEC, not elsewhere classified.

While all reported and available health expenditures were extracted at every hierarchical level of the SHA framework HC and HP categories, estimates were made to the second hierarchical level. This is due to the large amount of missing values at the third level categories, as will be presented in the results. Additionally, quality of health expenditures reported prior to 2000 has been highlighted as questionable by previous research and while all extracted data are made available since 1995, estimation of the HC and HP categories is presented from 2000 to 2017.^11 25^

### Measuring health expenditure trends

Using the estimated health expenditures by HC and HP categories, we estimated the relationship between these expenditures and GDP. Employing a generalised additive model, we estimated how the share of total health expenditures within HC and HP categories change as GDP per capita increased after controlling for trends in time.^26^ This approach is in line with previously published research and additional model specifications can be found in the online supplemental appendix 1.^16^

All statistical estimations were conducted using R V.5.3.3. The Bayesian multivariate model was performed using Bayesian Regression Models using Stan and the generalised additive models used the mgcv package.^26 27^

### Total health expenditure and covariate data

Health expenditures are modelled as a share of total health expenditure, which is sourced from annual work published by Global Burden of Disease Health Financing Collaborator Network and exclude capital formation.^1^ This same source is used for estimates of GDP and the share of total health expenditures from government sources. Other independent variables used for this analysis were sourced from the Global Burden of Disease and can be found on the Global Health Data Exchange website.^28^ These covariates are the Health Access and Quality Index, the average number of years of maternal education, the total fertility rate, age-standardised female HIV prevalence and proportion of population living in urban areas.^29–31^

### Patient and public involvement

As this study relied on secondary data, patients and the public were not involved in the formulation of this research.

## Results

### Compiled health expenditures

**Figure 1:**
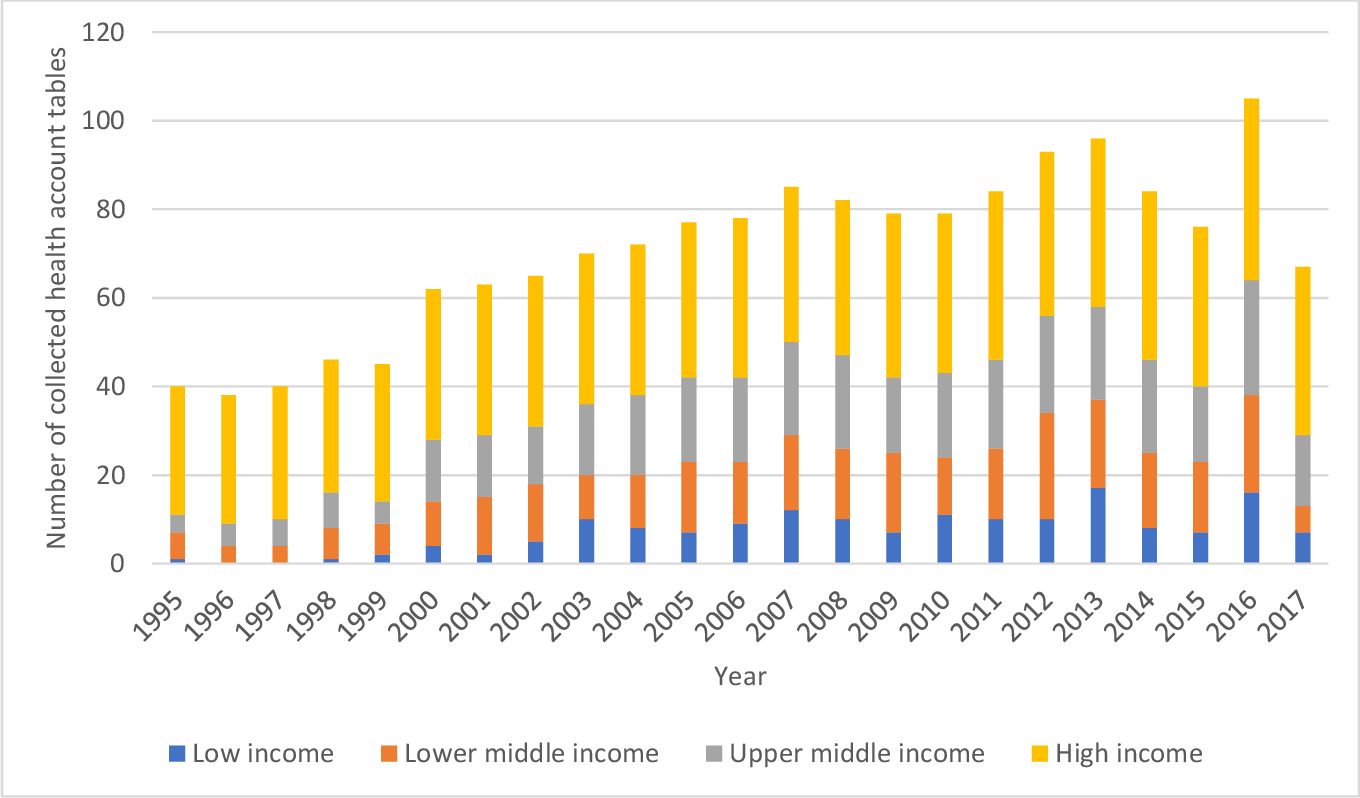
Collected National Health Accounts by World Bank income groups. This presents the number of National Health Accounts collected and used for this research by year and by World Bank income group.

A total of 1662 country-years worth of health accounts were identified, extracted, cleaned and standardised. Of the 1662 country-years of data, 1256 disaggregate expenditures by HC, 1113 by HP and 840 by the cross-classification of both. These data span 148 countries and contain information between 1995 and 2017. Figure 1 presents the number of country-years reported over time by World Bank income groups. Health expenditures reported since 2000 make up the majority of the collected data (86% or 1391 country-years). Just under 50%, or 799 country-years, of health expenditure data are from high-income countries, while middle-income and low-income countries account for the remaining 663 and 164, respectively. Over time, more low-income and middle-income countries reported HC and HP data. Twenty-eight countries have data for all years; of which, all but one (Turkey) are high-income countries. The median number of years a country reported data for is 19. Of 195 countries recognised by the United Nations, this analysis identified 55 countries where no health account data were available (online supplemental appendix table A.3).

### Expenditures by HC and HP

After cleaning the extracted health expenditures and standardising HC and HP categories between SHA versions, there were 91 HC categories and 74 HP unique categories. For utility of this research and applicability to policy topics, these categories were limited to 41 HC and 21 HP categories. The categories kept are the most reported by countries, while those dropped were only subcomponents of retained categories (online supplemental appendix table A.4). Even with a reduced number of categories, the most complete matrix of health expenditures still has 861 unique HC and HP cross-classifications. The most complete HC by HP matrix collected had expenditures for 52% of these 861 cells, while on average only 8% of all cells were in country-reported matrices. While most of the reported cross-classifications were relatively incomplete, total values for first, second, and third levels of HC and HP categories were reported at a significantly higher rate. Online supplemental appendix figure A.1 shows that over time reporting improved of all HC level totals (meaning not disaggregated by HP categories) with over 60% of all first level totals being reported in 2014, 2016 and 2017. This same improvement over time was observed for HP totals (meaning not disaggregated by HC categories) although there is a time lag for most recent years in reporting. As significant an improvement was not observed for reported expenditures of non-total HC and HP categories, which constitutes the majority of missing data was found.

In total, 110 070 data points of health expenditures reported by the 41 HC and 21 HP categories were identified and collected from health accounts. A complete list of HC and HP categories and the number of data points collected can be found in the online supplemental appendix table A.4.

### Estimated health expenditures by HC

**Figure 2:**
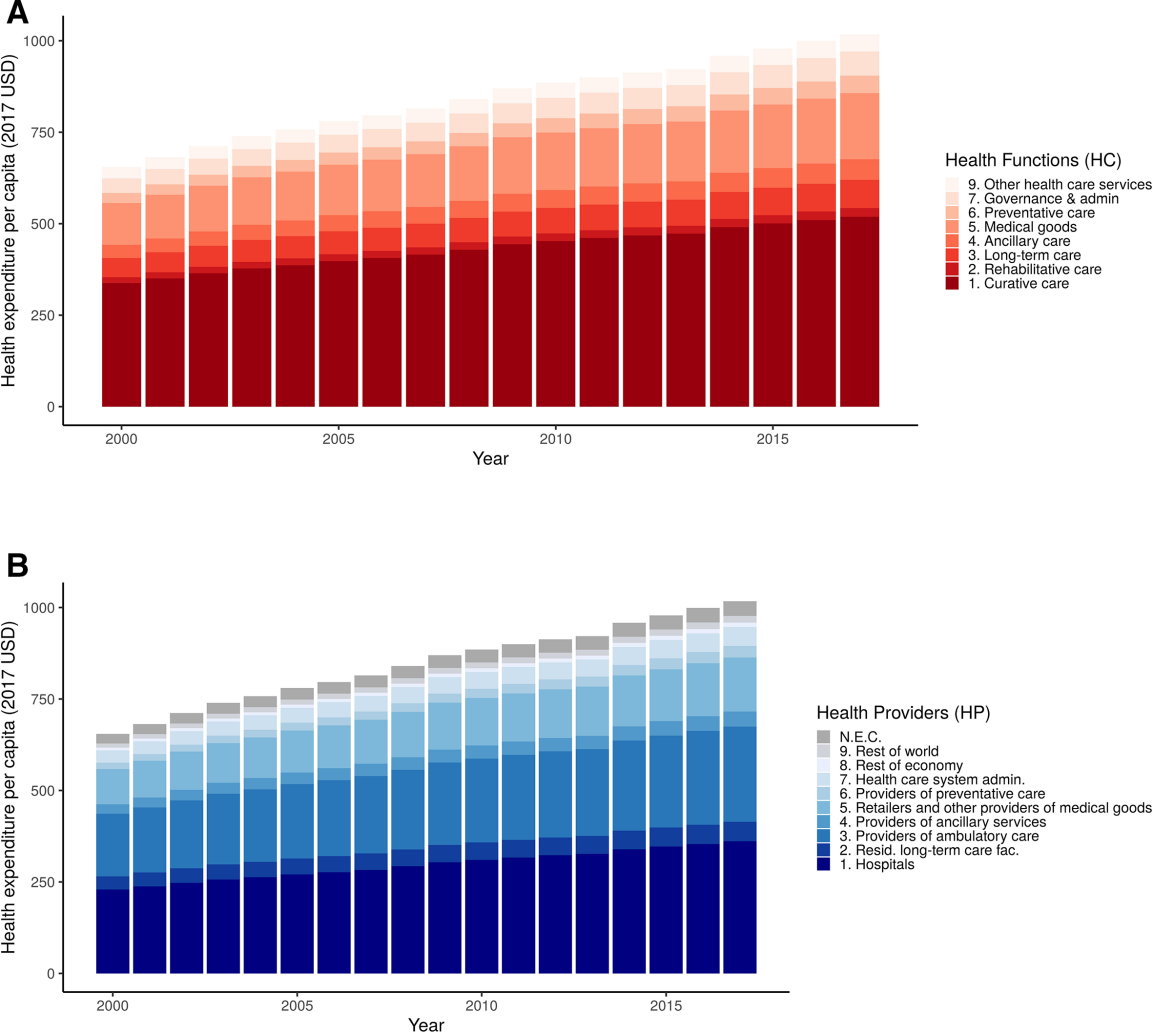
Globally estimated health expenditures per capita by health functions and health providers. (A) The composition of total health expenditures by health functions, as defined by the System of Health Accounts, between 2000 and 2017. (B) The composition of total health expenditures by health providers, as defined by the System of Health Accounts. Expenditures are presented in 2017 US dollars per capita. NEC, not elsewhere classified. Resid. long-term care fac., Residential long-term care facilities.

**Figure 3:**
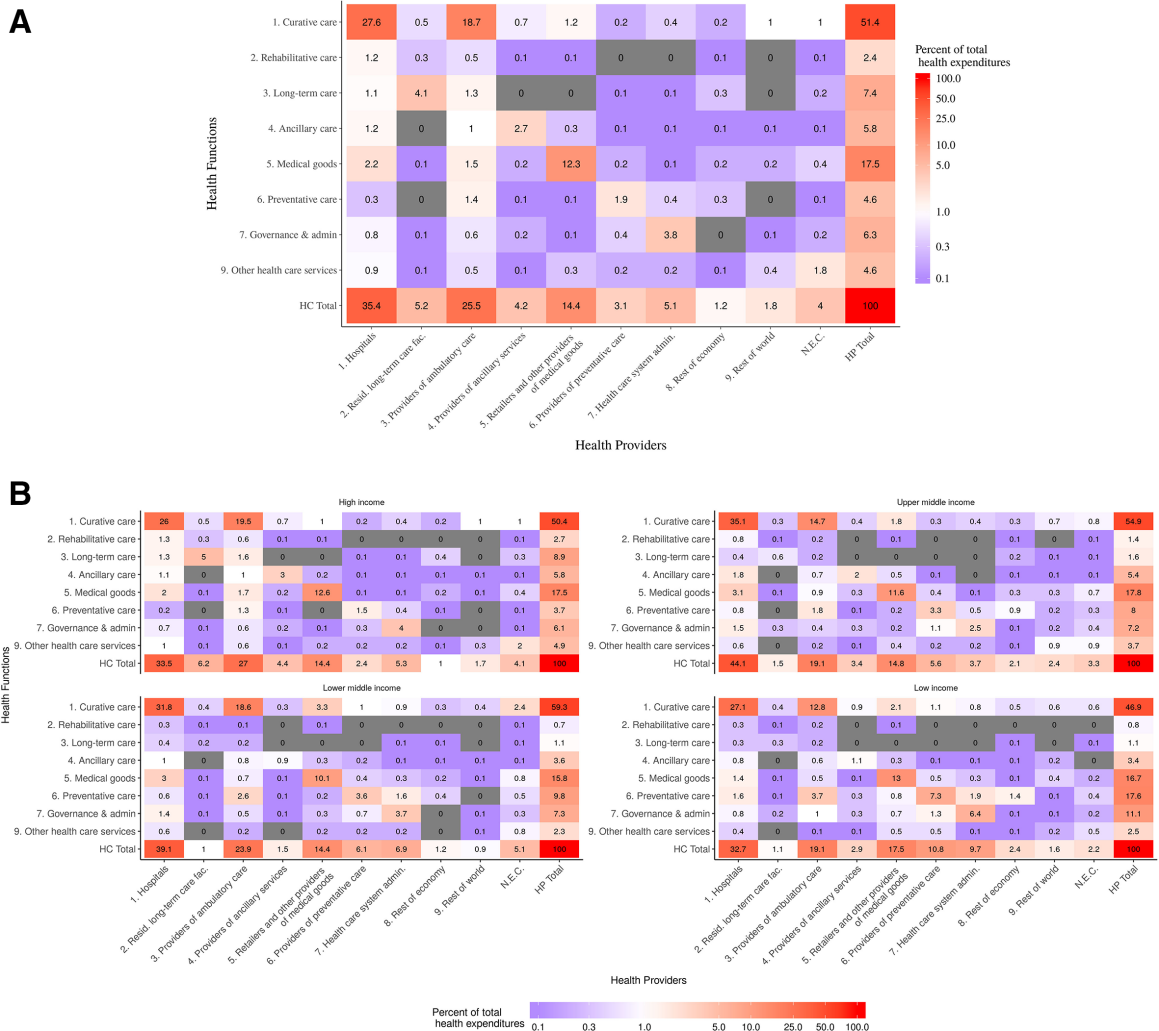
Health spending by health functions and health providers as a percent of total health expenditures, 2017. (A) Estimated global health expenditures by the cross-classification of health functions (HC) and health providers (HP) in 2017. All numbers are presented as a share of total health expenditures. (B) Further breakdown of total health expenditures by World Bank income group. NEC are expenditures not elsewhere classified by the System of Health Accounts framework. Resid. long-term care fac., Residential long-term care facilities.

Figure 2A and figure 3A show that among HC, curative care and medical goods make up the two largest shares of estimated total global health expenditures, representing 51.4% (UI between 33.2% and 59.4%) and 17.5% (UI between 13.0% and 26.9%) of total health expenditures in 2017, respectively. As shown in table 1, curative care further can be disaggregated into inpatient, outpatient, day and home-based care which make up 21.9% (UI between 15.3% and 27.2%), 22.8% (UI between 13.5% and 28.6%), 1.9% (UI 1.0% to 3.6%) and 0.6% (UI between 0.3% and 0.5%) of total health expenditures during this same time period, respectively. The category of medical goods breaks into pharmaceuticals and other medical non-durable goods, therapeutic appliances and prosthetics, and NEC goods which represent 13.4% (UI between 9.0% and 21.0%), 2.3% (UI between 1.5% and 4.1%) and 1.8% (UI between 0.5% and 4.6%) of total health expenditures, respectively. Table 1 presents these findings in 2017 USD.

As can be seen in figure 3B, across World Bank income groups, high-income countries spent 8.9% (UI 6.4% to 14.0%) or $445 per person (UI $322 to $700) of total health expenditures on long-term care, far more than the next closest group, upper middle-income countries, at just 1.6% (UI 0.7% to 4.4%) or $7.6 per person (UI $3.2 to $20.9). Low-income countries spent more on preventive care (17.6%) as a share of total health expenditures than any other income group, with lower middle-income, upper middle-income, and high-income countries spending 9.8%, 8%, and 3.7%, respectively. Fairly consistently, country income groupings spent between 15.8% and 17.8% of all health expenditures on medical goods. Interestingly, low-income countries spent more than any other income group on governance and administrative services (HC 7) at 11.1% of total expenditures.

**Figure 4:**
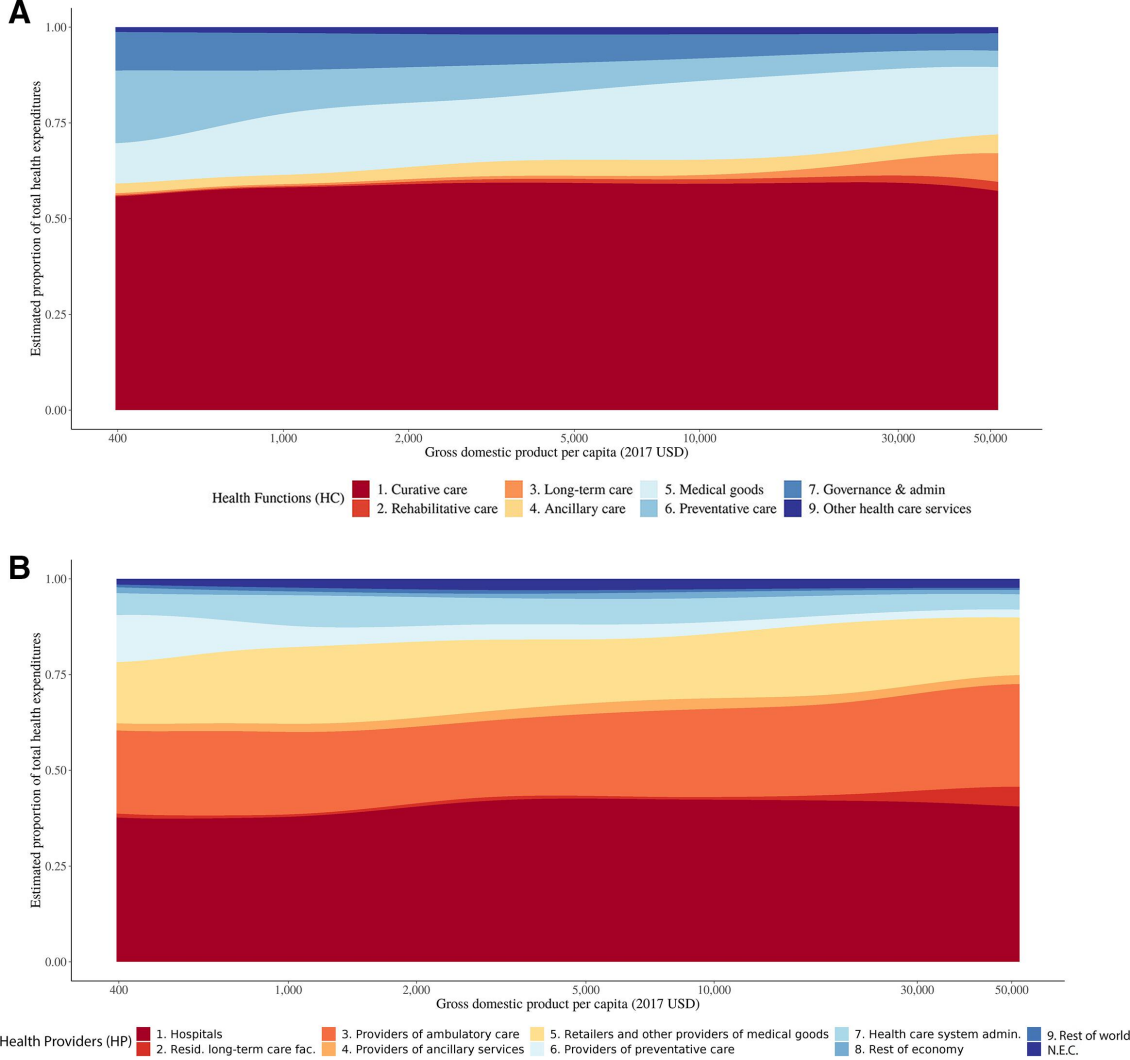
Estimated relationship between global health expenditures and gross domestic product. (A) The estimated relationship between the composition of global health expenditures by health functions and gross domestic product. (B) The estimated relationship between the composition of global health expenditures by health providers and gross domestic product. Health function and provider categories are based on the System of Health Accounts categories. These relationships are estimated using a generalised additive model. NEC, not elsewhere classified.

Figure 4A presents the relationship between estimated health functions as a share of total health expenditures with GDP per capita. Across all possible values of GDP per capita, curative care makes up the majority of health expenditures. As GDP increases, preventive care and administration (on average) make up a smaller share of total health expenditures. Long-term care expenditures increase with GDP, especially countries with more than $10 000 per person.

### Estimated health expenditures by HP

Figure 2B and the bottom row of figure 3B show health expenditures by providers over time and in 2017. Over three-quarters of the estimated global health expenditures by health providers fell into hospital providers (35.4%, UI 30.3% to 38.9%), providers of ambulatory care (25.5%, UI 21.1% to 28.8%) and retailers of medical goods (14.4%, UI 12.4% to 16.3%). Within hospital providers, as shown in table 1, general hospitals made up the largest component of health expenditures, with 23.9% (UI 17.6% to 29.8%) of global total health expenditures. Within providers of ambulatory care, medical practices and ambulatory healthcare centres made up 8.1% (UI 5.1% to 11.2%) and 6.1% (UI 3.58% to 8.7%) of total health expenditures, respectively. Table 1 presents these findings in 2017 USD.

Across income groups, as seen in the bottom rows of the matrices in figure 3B, low-income and high-income countries spent a similar share of total health expenditures in hospitals, 32.7% (UI 28% to 37.5%) and 33.5% (UI 27.6% to 37.6%), respectively. Lower middle-income countries spent 39.1% (UI 27.1% to 52.8%) and upper middle-income countries spent 44.1% (UI 29.9% to 54.2%) of total health expenditures in hospitals. High-income countries spent the most of any income group within providers of ambulatory care, 27.0% (UI 21.1% to 30.9%) of total health expenditures, while upper middle-income and low-income countries spent just over 19% in the same settings. Similar to healthcare functions, low-income countries spent more of their health expenditures than any other country income group within providers of preventive care, at 10.8% (UI 3.1% to 7.9%).

### Estimated health expenditures by cross-classification of HC and HP

Figure 3A presents the estimated share that each cross-classification of HC and HP, at the highest levels, made up of global total health expenditures in 2017. Curative care in hospital settings made up the largest share of total health expenditures at 27.7% (UI 9.2% to 37.8%), followed by curative care from providers of ambulatory care with 18.6% (UI 5.4% to 27.1%) and medical goods provided by retailers of medical goods with 12.4% (UI 5.6% to 17.1%).

Figure 3B highlights differences between the cross-tabulation of HC and HP expenditures in 2017 across World Bank income groups. We can see that not only do low-income countries spend more on preventive care, but that more is provided in the hospital setting than any other income group. Also, low-income countries spent a similar share of expenditures on medical goods as other income groups, however, they spent less in the hospital settings and more in retailers of medical goods and providers of ambulatory care.

## Discussion

National Health Accounts are used by national and international policymakers and researchers to understand the flow of expenditures for healthcare services and providers to make course corrections and set national and global goals, such as the Sustainable Development Goals and reaching Universal Health Coverage.^32 33^ Unfortunately, no single resource has compiled these country-reported health expenditures, let alone attempted to estimate the admittedly large amount of missing data. This research sought out and collated 1662 country-years of health expenditure estimates from country-reported health accounts categorised into HC and HP.^4 9^ It identified 55 countries that have no HC and HP estimates between 1995 and 2017. For country-years that do have expenditures with HC and HP information, these details are often incomplete and/or worsen the more granular the HC and HP categorisations become. These gaps limit the ability of policymakers and researchers to conduct the intended analyses within and across countries. We used a Bayesian multivariate statistical analysis leveraging all collected expenditures across countries and years to estimate a complete time-series of expenditures with uncertainty.

Policymakers and researchers can now access the country-reported data, as well as the complete set of country estimates of health expenditures broken down by the cross-classification of HC and HP to gain insights into past allocations. We found that across all levels of GDP, curative care made up more than half of all health expenditures. Low-income countries spent more, as a share of total health expenditures, on preventive care and governance and administration. High-income countries spent 4.5 times more on long-term care than upper middle-income countries. Across income groups, countries spent a relative similar proportion of the expenditures on medical goods. Of health providers, hospitals were estimated to comprise 35.4% of global health expenditures in 2017.

In this time of focus on epidemic preparedness due to the novel coronavirus pandemic, we highlight that higher income countries spent a smaller percentage of their health expenditures on preventive care than lower income countries. According to SHA guidelines, preventive care includes *early disease detection programmes* (HC 6.3), *epidemiological surveillance and risk and disease control programmes* (HC 6.5), and *preparing for disaster and emergency response programmes* (HC 6.6).^4^ In fact, it is estimated that high-income countries spent only 0.6% of health expenditures on these three categories in 2017, while lower middle-income and low-income countries spent an estimated 2.0% and 2.2%, respectively. In absolute terms, high-income countries still spent significantly more on these areas of prevention. Globally, these preventive care expenditures were provided mostly by preventive care, ambulatory care and hospital providers. As the world adapts to the impact of the novel coronavirus pandemic, it will be a point of great interest to see how expenditures on epidemic preparedness change across countries and providers in both absolute and relative terms.

While National Health Accounts provide the best attempt at capturing consistently reported health expenditures across countries, they are limited by underlying data differences and availability. Reasons for these differences include challenges in disentangling expenditures to map directly to the SHA categories, differences or a complete lack of sources for certain categories of health expenditures, and actual differences in country-specific health systems.^10 34^ This research attempts to model across countries, time and categories of spending to continue to push forward the comparability of these health expenditures, but the underlying differences in reporting may still affect the interpretation of these numbers and necessitate additional details to more accurately use these estimates when comparing across countries.

High-income countries made up the majority of the collected HC and HP expenditures. While low-income and middle-income countries have increased their reporting of these expenditures in recent years, the estimates produced, especially in earlier years and where no data were available, are potentially biased toward observed trends from high-income countries. For this reason, the estimates adjust for differences in demographics, development, and healthcare access and UIs are presented for every estimate of this research. We hope these estimates provide policymakers and researchers an opportunity to explore changes in health expenditure allocation over time, as policies change, and as they relate to population health.

## Conclusion

Health expenditures disaggregated by types of services and providers are often unavailable, missing, or not comparable across countries and time; thus, this essential information is unavailable to provide policymakers and researchers a holistic understanding of how expenditures have been used and if adjustments should be made. This research aggregated reported health expenditures by HC (services) and HP from all available National Health Accounts. Collating and assessing the completeness of these raw data highlight the difficulty in its collection, reporting and usability. While stakeholders continue to call for continued improvement in the reporting and collection of these disaggregated health expenditures, the need to fill in the current gaps is undeniable.^13 15 32^ Using all these newly collated data within a statistical approach leveraging correlates, such as national income and education, allowed for estimates, with uncertainty, to be calculated to fill this information gap. These data and estimates will allow national and international stakeholders to understand past trends in health expenditures and be used as one piece of information to help inform adjustments and course corrections to best reach national and international health targets.

## Data Availability

Data are available in a public, open access repository. The data that support the findings of this study will be made publicly available at IHME’s Global Health Data Exchange (GHDx) website (http://ghdx.healthdata.org/) upon publication.
