# Supplementary material for: Health expenditures by services and providers for 195 countries, 2000–2017
Source: BMJ Glob Health. 2021 Jul 30;6(7):e005799. doi: 10.1136/bmjgh-2021-005799 (PMC8327839; doi:10.1136/bmjgh-2021-005799)
Supplement: Supplementary data [file bmjgh-2021-005799supp001.pdf]

Appendix

Contents

Appendix 1
A.1 Health Account Search Terms Process 2
A.2 Health Expenditures Detailed Extraction Process 2
A.3 Limiting complexity of health expenditure HC and HP matrix 3
A.4 Additional details of estimation of complete time-series 3
A.5 Estimation of health expenditures 4
Method comparison 4
A.6 Estimating the relationship between gross domestic product and the composition of total health expenditures by functions and providers (Figure 6 in article) 4
Table A.1. Estimation method validity tests, root mean squared errors 5
Table A.2. Health Function Transition Between System of Health Accounts Versions 5
Figure A.1 Completeness of collected national health account expenditures by health function and health provider expenditures by year 8
Figure A.2 Health expenditures by World Bank income groups 10
Table A.3. Countries without any available NHA healthcare function or healthcare provider expenditures 11
Figure A.3 Recreation of manuscript figures 2 and 3 with only countries which had NHA healthcare function (HC) or healthcare provider (HP) expenditures 12
Table A.4. Collected Data by Health Functions (HC) and Health Providers (HP) – sorted by number of data points 15

A.1 Health Account Search Terms Process

Keyword searches using Google search engine included the following search terms:

“National Health Accounts”

“Health Accounts”

“National Health Expenditures”

Health account collection flow

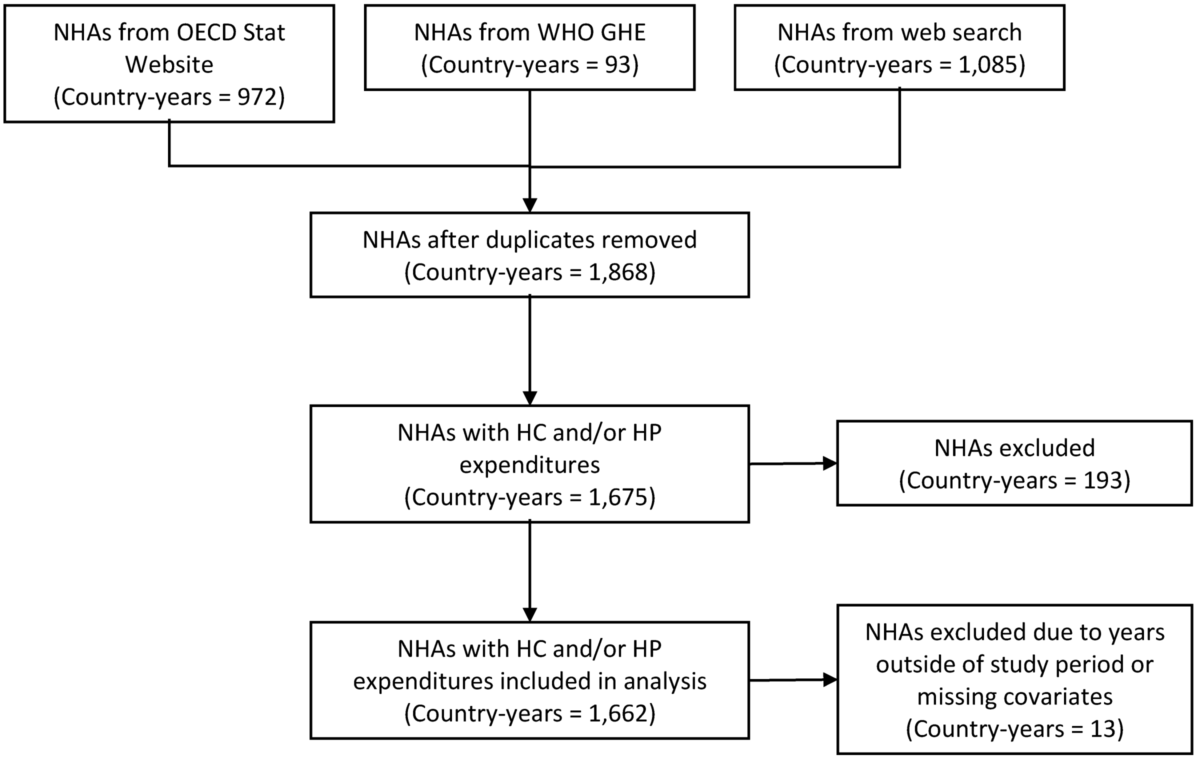

A.2 Health Expenditures Detailed Extraction Process

If the data were already assessable via an excel table or similar format, they were simply compiled and vetted for consistent reporting with their respective SHA guide, 2000 or 2011.(4,9) Those health expenditure data that were only found in their report form, were either extracted using PDFtoExcel or by hand.

A.3 Limiting complexity of health expenditure HC and HP matrix

The SHA 2011 HC and HP categories have 51 and 70 hierarchical categories, respectively. When considering these two sets of categories in a cross tabulated format, this produces over 3,500 unique combinations for 4,480 country-years of health expenditure data. The need to estimate over 15 million data-points is not a feasible goal given the amount of missing information, the computational power needed, and the fact that not every combination of health function and health provider exist in a health system.

A.4 Additional details of estimation of complete time-series

After extraction, cleaning, compiling, and reconciling differences of all identified NHA health expenditures reported by HC and HP categories, missing cross-tabulations of these data and countries with no data were estimated using multiple statistical approaches. These include a linear mixed effects model, Bayesian multivariate model, and spatial temporal Gaussian process regression (ST-GPR).(22–24) All three estimation methods use the same dependent variable – the reported health expenditure for each cross-tabulation of HC and HP as a share of total health expenditure for that given country-year. The dependent variable is then logit transformed to remain bound between 0 and 1. The linear mixed effects and Bayesian multivariate models allow all data to be estimated within one model specification represented in Equation 1 below.

Equation 1: $$y_{c,t,i,j} = X_{c,t} + s_t + \alpha_i + \alpha_j + \tilde{\alpha}_c + \tilde{\alpha}_{i,j} + \tilde{\alpha}_{c,i,j} + \varepsilon_{c,t,i,j}$$

Where *c* = country, *t* = years, *i* = NHA health functions categories, *j* = NHA health provider categories. $y_{c,t,i,j}$ are country-year reported health spending by NHA categories (*i,j*) as a share of total health expenditures. $X_{c,t}$ are country and year varying covariates (fraction of total health expenditures that is from government, health access and quality index, GDP per capita, urbanicity, total fertility rate, years of maternal education, female HIV prevalence), s_t is a penalized spline over time, α_i are health function random-slopes (fixed effects), α_j are health provider random-slopes (fixed effects), $\tilde{\alpha}_c$ are country random intercepts (random effects), $\tilde{\alpha}_{i,j}$ are health function and provider category interaction random intercepts (random effects), and $\tilde{\alpha}_{c,i,j}$ are random intercepts (random effects) of the interaction between country and health function and provider interactions.

However, ST-GPR cannot allow for random or fixed effects by HC and HP categories and thus is unable to differentiate between the hierarchical complexity of the cross tabulation of function and provider categorization. Thus the model had to be fit for each cross-tabulation of HC and HP categories. Briefly, the linear mixed effects and Bayesian multivariate models utilize nearly the exact formulation, with the exception of the Bayesian method contains a semi-parametric relationship between time and our outcome variable using penalized splines, instead of a linear relationship. While the model specifications between the linear mixed effects and Bayesian models remain similar, the difference between the maximum likelihood estimation versus the Bayesian incorporation of weakly informative priors and Hamiltonian Monti Carlo sampler to estimate the posterior distribution of our parameters leads to differences in estimates, specifically for cross-tabulations without many observations.

ST-GPR consists of three parts, the first is a linear mixed effects model which produces first-stage predictions. These first stage predictions are then used in the second step to estimate spatiotemporal

patterns using weights to average the residuals producing spatiotemporally smoothed predictions. The final step uses a Gaussian process regression with the raw data and the smoothed predictions as the mean function to produce final ST-GPR predictions.(24) Again, ST-GPR was estimated for each cross-tabulation of HC and HP categories across countries and years. This allows us to test if individual models per cell of the HC and HP matrix produce better estimates than a single model across all collected data.

A.5 Estimation of health expenditures

Method comparison

Table A1 (below) presents the root mean squared errors (RMSE) for both in- and out-of-sample predictive validations. The RMSEs were calculated in both per capita and as a share of total health expenditures. The lower the RMSE the better the predictions relative to the data. Using the three estimation methods (linear mixed effects regression, Bayesian regression models in Stan (BRMS), and ST-GPR) in-sample validity tests showed that the BMRS and mixed effects methods had comparable results (showing that the estimates were on average 3.3 percent from the data), while ST-GPR performed significantly worse (12.8 percent from the data). Based upon an out-of-sample validity test where 20% of the collected data was randomly dropped, the models were fit to the remaining 80% of the data, predictions were made for all country-years and HC and HP categories, and the RMSEs were calculated between the predictions and the randomly dropped 20% of data, it was found that the mixed effects slightly out performed BRMS, while ST-GPR again was significantly worse than the other two. Another out-of-sample validity test conducted was dropping complete data for certain countries (examples provided in Table 2 for Australia and Thailand) to mimic the lack of all data for a country. In this last out-of-sample validity test, it was found that BRMS outperformed the other two methods. In addition to these statistical cross-validation differences, it was observed that BRMS estimated less erratic trends over time within health function and provider categories that lacked data, as compared to the mixed effects model and ST-GPR.

ST-GPR would not allow for the complete dataset to be estimated in one model as it does not differentiate different categories for the same country-year. As such ST-GPR instead estimated 759 independent model runs, one for each cross tabulation of HC and HP with data. The inability to borrow strength from values of related HP and HC categories meant that some of these models attempted to fit with very few data points and were unable to converge. As such, ST-GPR is unable to produce a complete time-series for all HC and HP cross tabulations of interest. Of the 759 models with collected data, only 606 categories of HC and HP cross tabulations were able to converge.

A.6 Estimating the relationship between gross domestic product and the composition of total health expenditures by functions and providers (Figure 6 in article)

Using the estimated complete time-series of country level health expenditures by health functions (HC) and health providers (HP), we used independent generalized additive model (GAM from mgcv package in R) to estimate the relationship between each HC and HP category as a share of total health expenditures (THE). The left hand side variables were center log-ratio transformation to constrain the estimates of the proportions to sum to 1. The right hand side variables consisted of logged gross domestic product (GDP) per capita and time (in years). These were estimated as non-linear relationships

using penalized splines, which identified the appropriate placement for the knots, and a REML smoothing parameter estimation method.

Table A.1. Estimation method validity tests, root mean squared errors

	RMSE of All Data (In-sample)		RMSE of Dropped 20% of Data (Out-of-sample)		RMSE when Australia Is Dropped (Out-of-sample)		RMSE when Thailand Is Dropped (Out-of-sample)	
Model	Per capita space	Share space	Per capita space	Share space	Per capita space	Share space	Per capita space	Share space
BRMS	43.63	0.0333	55.17	0.0588	114.71	0.0266	12.02	0.0568
Mixed Effects	43.37	0.0338	53.93	0.0556	122.53	0.0287	12.16	0.0574
ST-GPR	380.69	0.128	73.69	0.0674	373.85	0.0787	59.46	0.2783

Table A.2. Health Function Transition Between System of Health Accounts Versions

SHA 2011 codes	Description	SHA 1.0 codes
**HC.1**	Curative care	HC.1
**HC.1.1**	Inpatient curative care	HC.1.1
**HC.1.1.1**	General inpatient curative care	
**HC.1.1.2**	Specialised inpatient curative care	
**HC.1.2**	Day curative care	HC.1.2
**HC.1.2.1**	General day curative care	
**HC.1.2.2**	Specialised day curative care	
**HC.1.3**	Outpatient curative care	HC.1.3
**HC.1.3.1**	General outpatient curative care	HC.1.3.1
**HC.1.3.2**	Dental outpatient curative care	HC.1.3.2
**HC.1.3.3**	Specialised outpatient curative care	HC 1.3.3
**HC.1.4**	Home-based curative care	HC.1.4
**HC.2**	Rehabilitative care	HC.2
**HC.2.1**	Inpatient rehabilitative care	HC2.1
**HC.2.2**	Day rehabilitative care	HC2.2

**HC.2.3**	Outpatient rehabilitative care	HC2.3
**HC.2.4**	Home-based rehabilitative care	HC2.4
**HC.3**	Long-term care (health)	HC.3
**HC.3.1**	Inpatient long-term care (health)	HC.3.1
**HC.3.2**	Day long-term care (health)	HC.3.2
**HC.3.3**	Outpatient long-term care (health)	part of HC.3
**HC.3.4**	Home-based long-term care (health)	HC.3.3
**HC.4**	Ancillary services (non-specified by function)	HC.4
**HC.4.1**	Laboratory services	HC.4.1
**HC.4.2**	Imaging services	HC.4.2
**HC.4.3**	Patient transportation	HC.4.3
**HC.5**	Medical goods (non-specified by function)	HC.5
**HC.5.1**	Pharmaceuticals and other medical non-durable goods	HC.5.1
**HC 5.1.1**	Prescribed medicines	HC.5.1.1
**HC 5.1.2**	Over-the-counter medicines	HC.5.1.2
**HC 5.1.3**	Other medical non-durable goods	HC.5.1.3
**HC.5.2**	Therapeutic appliances and other medical goods	HC.5.2
**HC.5.2.1**	Glasses and other vision products	HC.5.2.1
**HC.5.2.2**	Hearing aids	HC.5.2.3
**HC.5.2.3**	Other orthopaedic appliances and prosthetics (excluding glasses and hearing aids)	HC.5.2.2
**HC.5.2.9**	All other medical durables, including medical technical devices	HC.5.2.4- HC.5.2.9
**HC.6**	Preventive care	HC.6, part of HC.R.4, HC.R.5
**HC.6.1**	Information, education and counseling programmes	Part of HC.6.9, part of HCR4, HC.R.5
**HC.6.2**	Immunisation programmes	Part of HC.6.3
**HC.6.3**	Early disease detection programmes	Part of HC.6.3, HC.6.4

**HC.6.4**	Healthy condition monitoring programmes	Part of HC.6.1, HC.6.2, HC.6.5
**HC.6.5**	Epidemiological surveillance and risk and disease control programmes	HC.6, part of HC. 4, HC. 5
**HC.6.6**	Preparing for disaster and emergency response programmes	Part of HC.6
**HC.7**	Governance, and health system and financing administration	HC.7
**HC.7.1**	Governance and Health system administration	HC.7.1
**HC.7.2**	Administration of health financing	HC.7.2
**HC.9**	Other health care services not elsewhere classified (n.e.c.)	

Figure A.1 Completeness of collected national health account expenditures by health function and health provider expenditures by year

Panel A. Health function totals

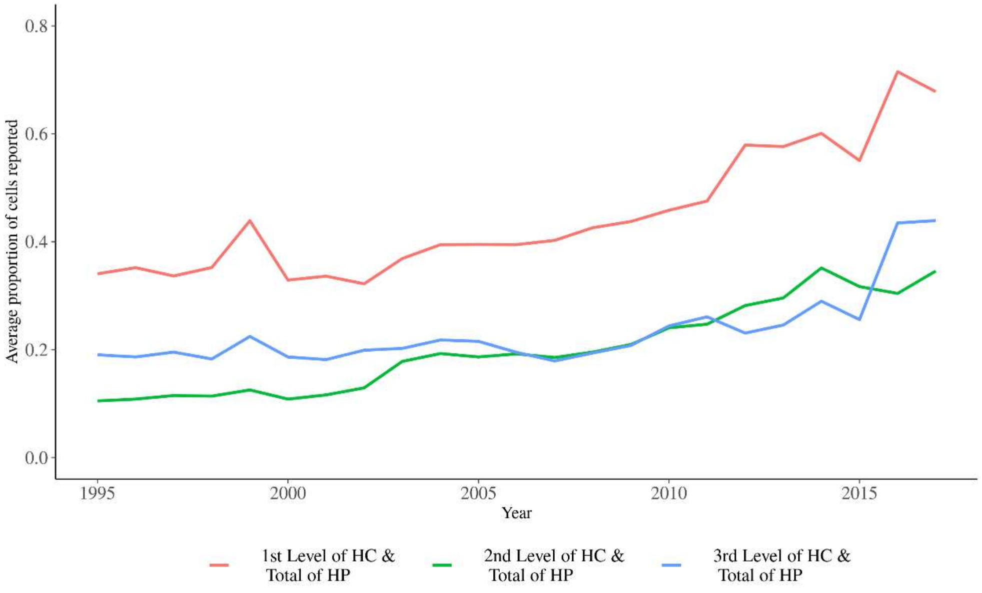

Panel B. Health provider totals

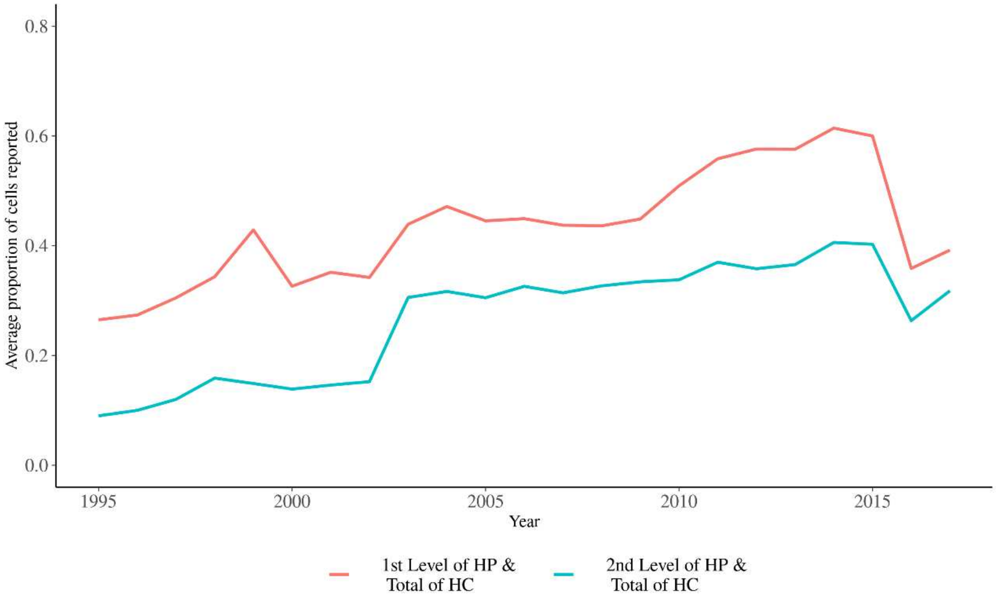

Panel C. Health function and health provider cross-tabulations

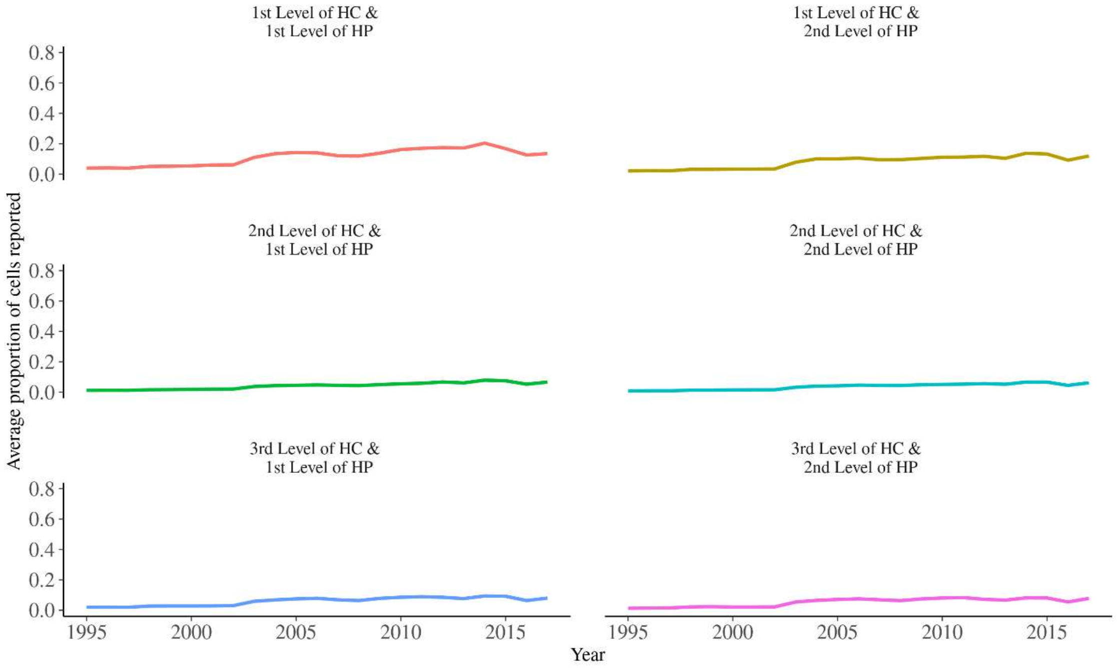

Note: Example of 1st Level is HC 5 (Medical goods) or HP 3 (Providers of ambulatory care); 2nd level is HC 5.1 (Medical goods - Pharms & other medical non-durable goods) or HP 3.3 (Providers of ambulatory care - Medical practices); 3rd Level is only for HC categories and an example is HC 5.1.1 (5.1.1 Medical goods - Prescribed meds)

Figure A.2 Health expenditures by World Bank income groups

Panel A. Health Functions

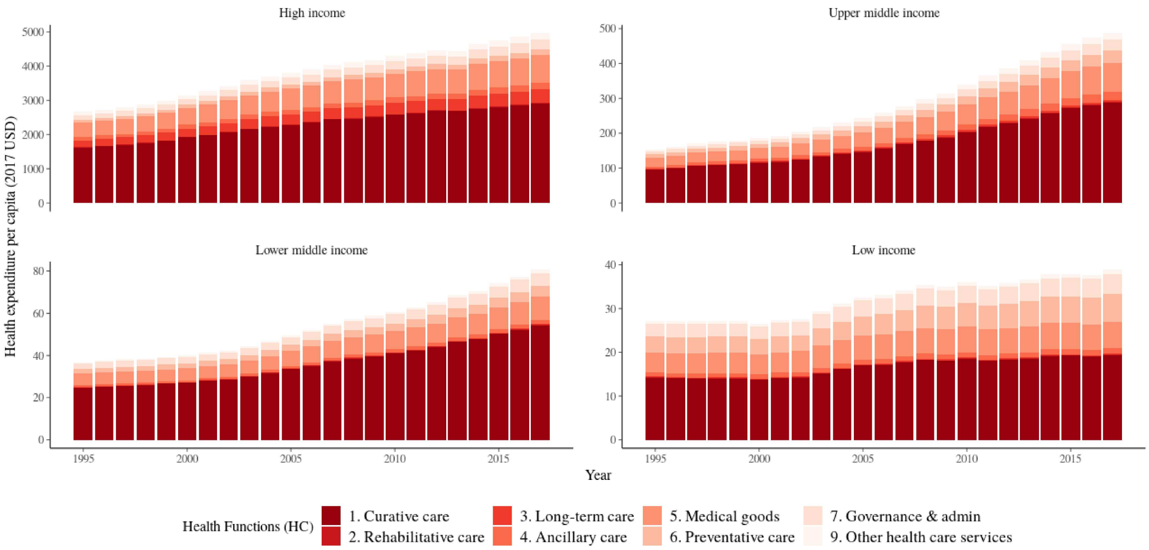

Panel B. Health Providers

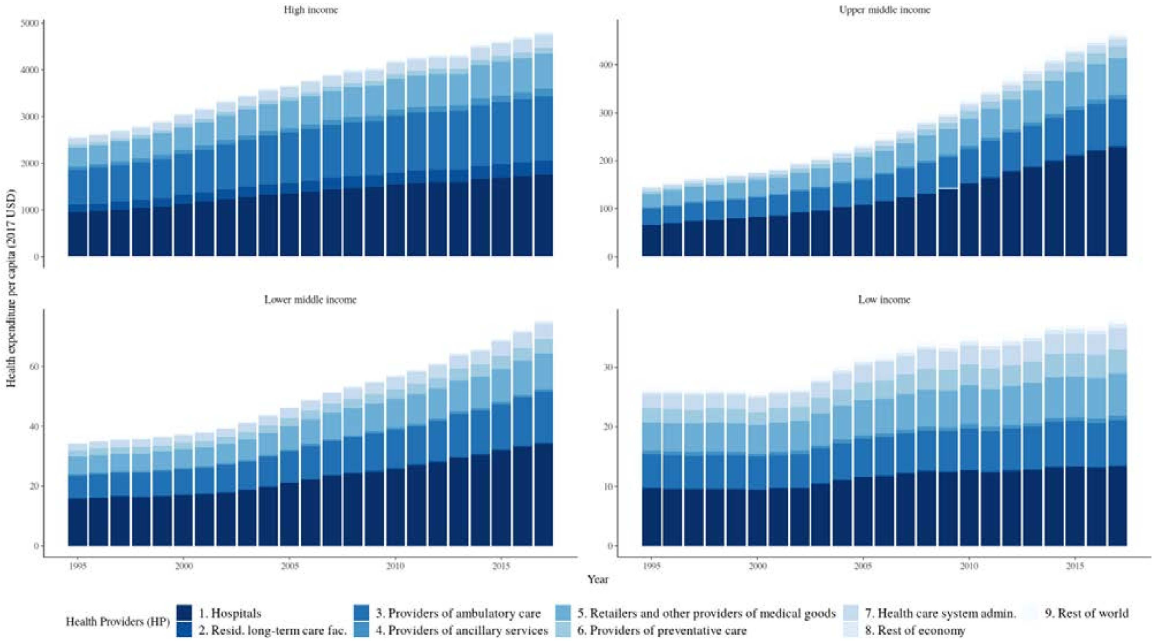

Table A.3. Countries without any available NHA healthcare function or healthcare provider expenditures

**ISO3**	**Country Name**
AGO	Angola
AND	Andorra
ARE	United Arab Emirates
ARG	Argentina
ASM	American Samoa
ATG	Antigua and Barbuda
AZE	Azerbaijan
BHR	Bahrain
BHS	The Bahamas
BLR	Belarus
BLZ	Belize
BRN	Brunei
CAF	Central African Republic
COL	Colombia
CUB	Cuba
CYP	Cyprus
DJI	Djibouti
DZA	Algeria
ERI	Eritrea
GNB	Guinea-Bissau
GNQ	Equatorial Guinea
GRD	Grenada
GRL	Greenland
GUM	Guam
HND	Honduras
HRV	Croatia
JAM	Jamaica
KWT	Kuwait
LBY	Libya
LCA	Saint Lucia
LSO	Lesotho
MHL	Marshall Islands
MKD	North Macedonia
MLT	Malta
MNP	Northern Mariana Islands
OMN	Oman
PAN	Panama
PER	Peru
PRI	Puerto Rico
PRK	North Korea
ROU	Romania
SAU	Saudi Arabia
SDN	Sudan
SGP	Singapore
SLB	Solomon Islands
SOM	Somalia
SSD	South Sudan
SWZ	eSwatini
SYR	Syria
TCD	Chad
TKM	Turkmenistan
UZB	Uzbekistan
VCT	Saint Vincent and the Grenadines
VEN	Venezuela
VIR	Virgin Islands

Figure A.3 Recreation of manuscript figures 2 and 3 with only countries which had NHA healthcare function (HC) or healthcare provider (HP) expenditures

To show that countries which had no reported health expenditures do not influence the main trends and findings presented in the main manuscript, all figures below include only countries which reported some data on healthcare functions and healthcare provider expenditures.

Recreated Figure 2 (with only counties with reported HC and HP expenditures) - Globally estimated health expenditures per capita by health functions and health providers

Panel A. Healthcare functions (HC)

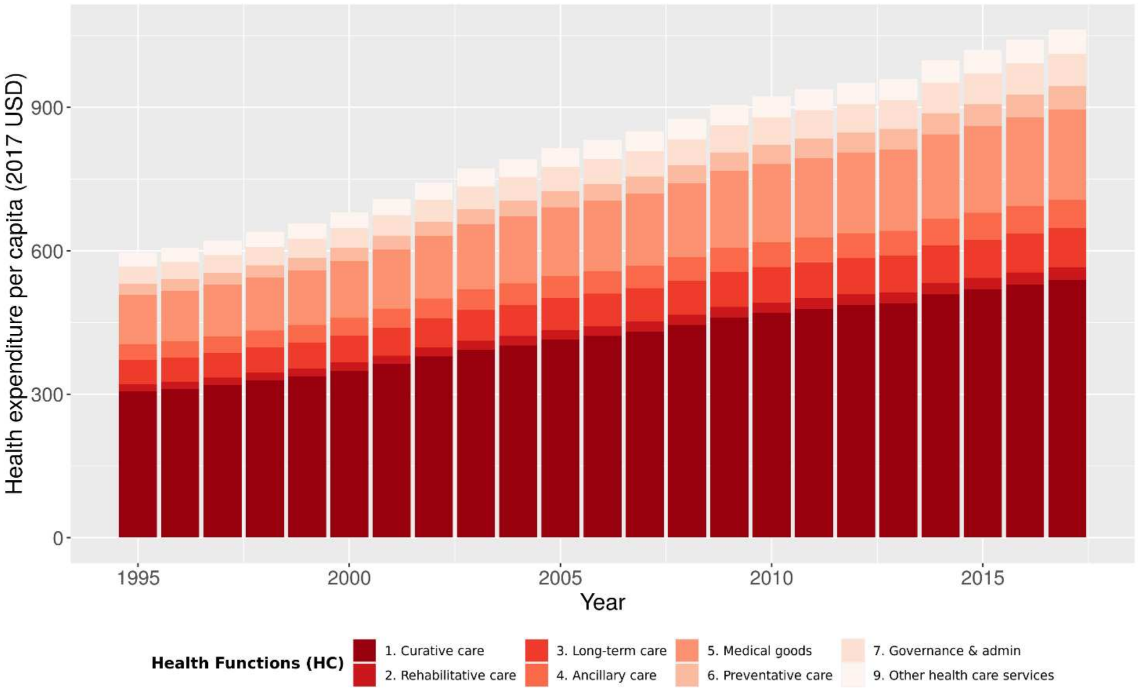

Panel B. Healthcare providers (HP)

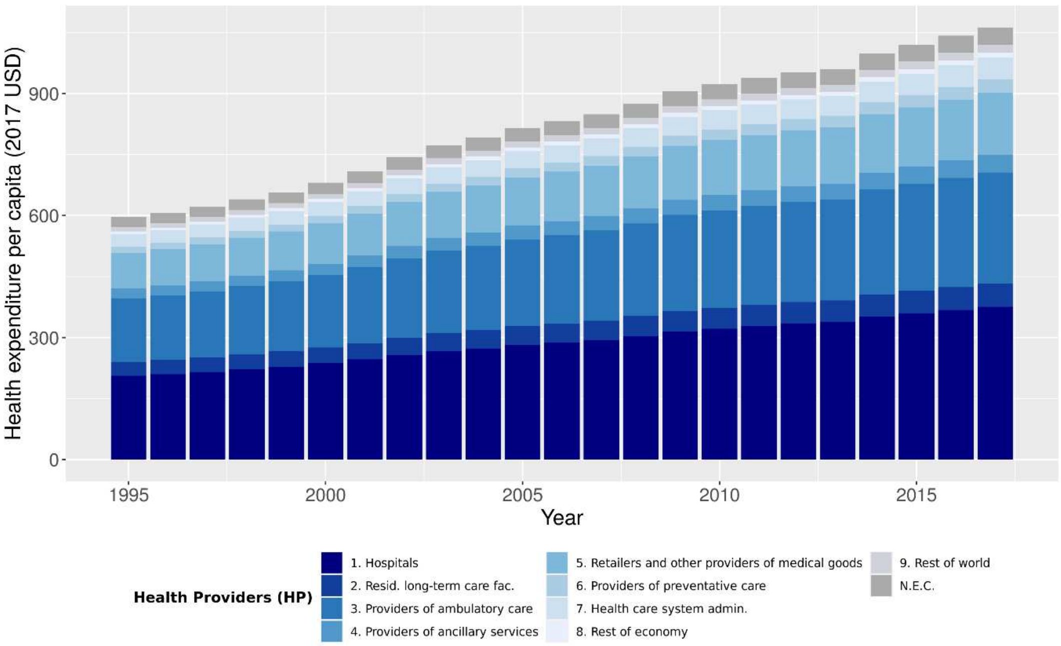

Recreated Figure 3 (with only counties with reported HC and HP expenditures) - Health spending by health functions and health providers as a percent of total health expenditures, 2017

Panel A.

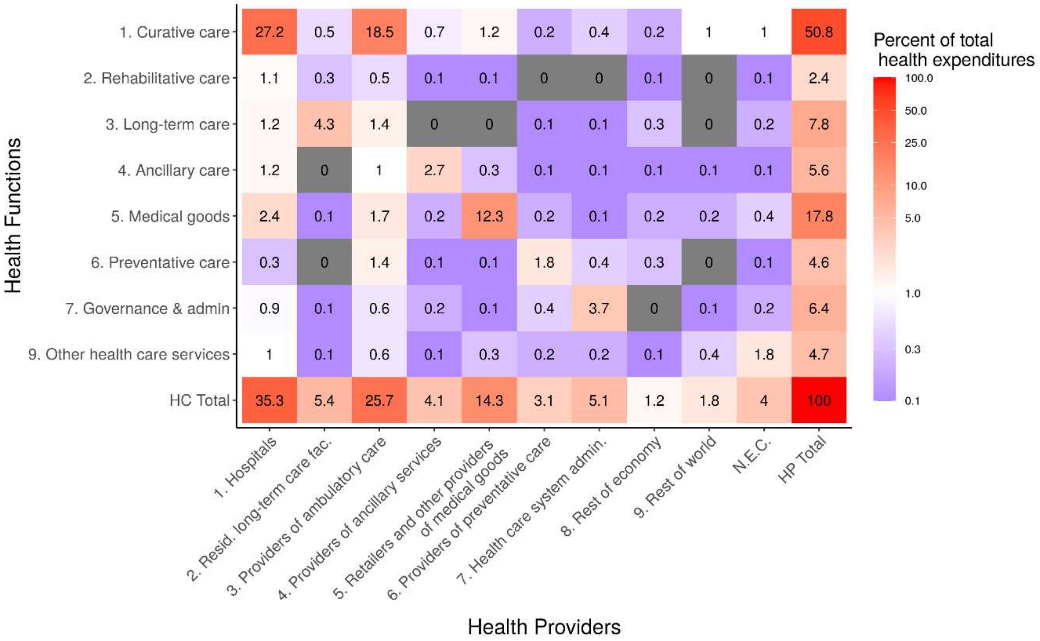

Panel B.

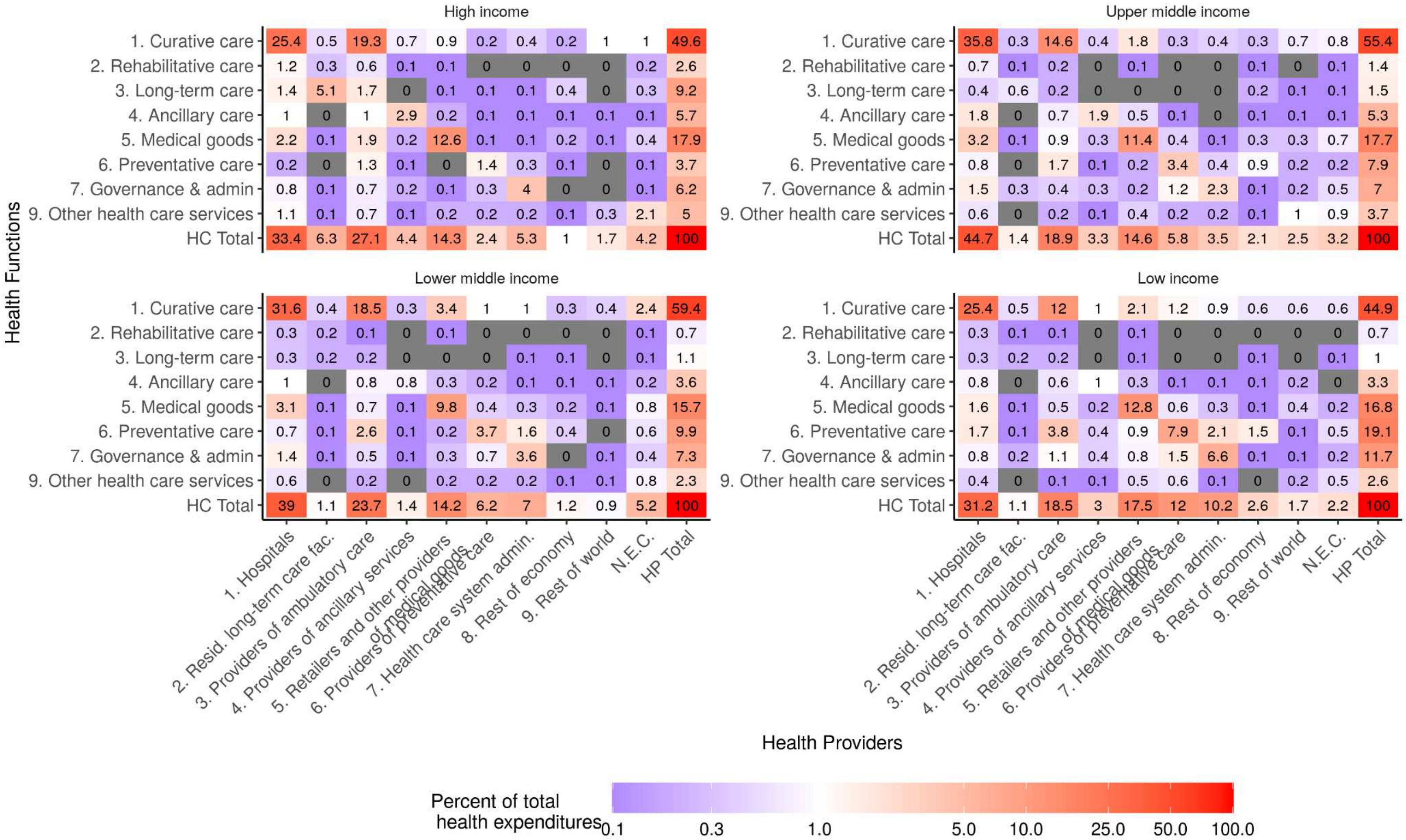
High income
Upper middle income
Lower middle income
Low income
Health Functions
1. Curative care
2. Rehabilitative care
3. Long-term care
4. Ancillary care
5. Medical goods
6. Preventative care
7. Governance & admin
9. Other health care services
HC Total
Health Providers
1. Hospitals
2. Resid. long-term care fac.
3. Providers of ambulatory care
4. Providers of ancillary services
5. Retailers and other providers of medical goods
6. Providers of preventative care
7. Health care system admin.
8. Rest of economy
9. Rest of world
N.E.C.
HP Total
High income
25.4 0.5 19.3 0.7 0.9 0.2 0.4 0.2 1 1 49.6
1.2 0.3 0.6 0.1 0.1 0 0 0 0 0.2 2.6
1.4 5.1 1.7 0 0.1 0.1 0.1 0.4 0 0.3 9.2
1 0 1 2.9 0.2 0.1 0.1 0.1 0.1 0.1 5.7
2.2 0.1 1.9 0.2 12.6 0.1 0.1 0.2 0.1 0.4 17.9
0.2 0 1.3 0.1 0 1.4 0.3 0.1 0 0.1 3.7
0.8 0.1 0.7 0.2 0.1 0.3 4 0 0 0.1 6.2
1.1 0.1 0.7 0.1 0.2 0.2 0.2 0.1 0.3 2.1 5
33.4 6.3 27.1 4.4 14.3 2.4 5.3 1 1.7 4.2 100
Upper middle income
35.8 0.3 14.6 0.4 1.8 0.3 0.4 0.3 0.7 0.8 55.4
0.7 0.1 0.2 0 0.1 0 0 0.1 0 0.1 1.4
0.4 0.6 0.2 0 0 0 0 0.2 0.1 0.1 1.5
1.8 0 0.7 1.9 0.5 0.1 0 0.1 0.1 0.1 5.3
3.2 0.1 0.9 0.3 11.4 0.4 0.1 0.3 0.3 0.7 17.7
0.8 0 1.7 0.1 0.2 3.4 0.4 0.9 0.2 0.2 7.9
1.5 0.3 0.4 0.3 0.2 1.2 2.3 0.1 0.2 0.5 7
0.6 0 0.2 0.1 0.4 0.2 0.2 0.1 1 0.9 3.7
44.7 1.4 18.9 3.3 14.6 5.8 3.5 2.1 2.5 3.2 100
Lower middle income
31.6 0.4 18.5 0.3 3.4 1 1 0.3 0.4 2.4 59.4
0.3 0.2 0.1 0 0.1 0 0 0 0 0.1 0.7
0.3 0.2 0.2 0 0 0 0.1 0.1 0 0.1 1.1
1 0 0.8 0.8 0.3 0.2 0.1 0.1 0.1 0.2 3.6
3.1 0.1 0.7 0.1 9.8 0.4 0.3 0.2 0.1 0.8 15.7
0.7 0.1 2.6 0.1 0.2 3.7 1.6 0.4 0 0.6 9.9
1.4 0.1 0.5 0.1 0.3 0.7 3.6 0 0.1 0.4 7.3
0.6 0 0.2 0 0.2 0.2 0.2 0.1 0.1 0.8 2.3
39 1.1 23.7 1.4 14.2 6.2 7 1.2 0.9 5.2 100
Low income
25.4 0.5 12 1 2.1 1.2 0.9 0.6 0.6 0.6 44.9
0.3 0.1 0.1 0 0.1 0 0 0 0 0 0.7
0.3 0.2 0.2 0 0.1 0 0 0.1 0 0.1 1
0.8 0 0.6 1 0.3 0.1 0.1 0.1 0.2 0 3.3
1.6 0.1 0.5 0.2 12.8 0.6 0.3 0.1 0.4 0.2 16.8
1.7 0.1 3.8 0.4 0.9 7.9 2.1 1.5 0.1 0.5 19.1
0.8 0.2 1.1 0.4 0.8 1.5 6.6 0.1 0.1 0.2 11.7
0.4 0 0.1 0.1 0.5 0.6 0.1 0 0.2 0.5 2.6
31.2 1.1 18.5 3 17.5 12 10.2 2.6 1.7 2.2 100
Percent of total health expenditures
0.1 0.3 1.0 5.0 10.0 25.0 50.0 100.0

Table A.4. Collected Data by Health Functions (HC) and Health Providers (HP) – sorted by number of data points

HC Category Name	HP Category Name	Number of Data Points Collected
HC Total	HP Total	1293
6. Preventative care	HP Total	1069
7. Governance & admin	HP Total	1060
5. Medical goods	HP Total	1020
HC Total	6. Providers of preventative care	981
HC Total	1. Hospitals	940
4. Ancillary care	HP Total	935
HC Total	3. Providers of ambulatory care	915
HC Total	5. Retailers and other providers of medical goods	915
HC Total	7. Health care system admin.	914
5.1 Medical goods - Pharms & other medical non-durable goods	HP Total	861
3. Long-term care	HP Total	792
1. Curative care	HP Total	767
1.3.2 Curative care - Outpatient - Dental	HP Total	750
1.3 Curative care - Outpatient	HP Total	743
6. Preventative care	6. Providers of preventative care	733
5.2 Medical goods - Therapeutic appliances & prosthetics	HP Total	732
1.1 Curative care - Inpatient	HP Total	712
5. Medical goods	5. Retailers and other providers of medical goods	698
7. Governance & admin	7. Health care system admin.	692
HC Total	8. Rest of economy	687
HC Total	2. Resid. long-term care fac.	686
2. Rehabilitative care	HP Total	685
3.1 Long-term care - Inpatient	HP Total	682

HC Total	3.1 Providers of ambulatory care - Medical practices	681
HC Total	4. Providers of ancillary services	679
HC Total	3.2 Providers of ambulatory care - Dental practices	651
1.3.1 Curative care - Outpatient - General	HP Total	643
HC Total	9. Rest of world	621
4. Ancillary care	4. Providers of ancillary services	614
HC Total	1.1 Hospitals - General	604
5.1 Medical goods - Pharms & other medical non-durable goods	5. Retailers and other providers of medical goods	597
5.2 Medical goods - Therapeutic appliances & prosthetics	5. Retailers and other providers of medical goods	585
1.3.3 Curative care - Outpatient - Specialized	HP Total	584
3.4 Long-term care - Home-based	HP Total	572
HC Total	3.3 Providers of ambulatory care - Other practices	570
1. Curative care	1. Hospitals	561
5.1.1 Medical goods - Prescribed meds	HP Total	557
1.3.2 Curative care - Outpatient - Dental	3.2 Providers of ambulatory care - Dental practices	557
1. Curative care	3. Providers of ambulatory care	556
6. Preventative care	3. Providers of ambulatory care	542
9. Other health care services	HP Total	537
HC Total	3.4 Providers of ambulatory care - Ambulatory health care centers	536
1.3 Curative care - Outpatient	3.1 Providers of ambulatory care - Medical practices	533
1.3 Curative care - Outpatient	3. Providers of ambulatory care	530
3. Long-term care	2. Resid. long-term care fac.	524
1.3.2 Curative care - Outpatient - Dental	3. Providers of ambulatory care	514
HC Total	3.5 Providers of home health care services	511

1.3 Curative care - Outpatient	3.2 Providers of ambulatory care - Dental practices	510
1.1 Curative care - Inpatient	1.1 Hospitals - General	509
1.1 Curative care - Inpatient	1. Hospitals	508
HC Total	1.2 Hospitals - Mental health	506
3. Long-term care	3. Providers of ambulatory care	496
1.3 Curative care - Outpatient	1.1 Hospitals - General	494
1.3 Curative care - Outpatient	1. Hospitals	490
3.1 Long-term care - Inpatient	2. Resid. long-term care fac.	489
HC Total	1.3 Hospitals - Specialized	483
1. Curative care	3.1 Providers of ambulatory care - Medical practices	483
1.3.1 Curative care - Outpatient - General	3. Providers of ambulatory care	476
2. Rehabilitative care	1. Hospitals	474
1. Curative care	3.2 Providers of ambulatory care - Dental practices	469
5.1.2 Medical goods - Over the counter meds	HP Total	464
1.3.nec Curative care - Outpatient - NEC	HP Total	463
1. Curative care	1.1 Hospitals - General	460
1.2 Curative care - Day	HP Total	454
2.1 Rehabilitative care - Inpatient	HP Total	453
6. Preventative care	8. Rest of economy	452
3.4 Long-term care - Home-based	3. Providers of ambulatory care	446
2. Rehabilitative care	3. Providers of ambulatory care	445
1. Curative care	3.3 Providers of ambulatory care - Other practices	443
5.1.1 Medical goods - Prescribed meds	5. Retailers and other providers of medical goods	443
2.3 Rehabilitative care - Outpatient	HP Total	442
5.1.3 Medical goods - Other medical non-durables	HP Total	439
3. Long-term care	3.5 Providers of home health care services	437

1.3.1 Curative care - Outpatient - General	3.1 Providers of ambulatory care - Medical practices	437
4. Ancillary care	1. Hospitals	431
1.4 Curative care - Home-based	HP Total	431
1.3 Curative care - Outpatient	3.3 Providers of ambulatory care - Other practices	428
3.4 Long-term care - Home-based	3.5 Providers of home health care services	417
6. Preventative care	1. Hospitals	409
3. Long-term care	1. Hospitals	405
1.3.3 Curative care - Outpatient - Specialized	3. Providers of ambulatory care	402
4. Ancillary care	3. Providers of ambulatory care	399
1.3 Curative care - Outpatient	3.4 Providers of ambulatory care - Ambulatory health care centers	399
1.1 Curative care - Inpatient	1.2 Hospitals - Mental health	395
5.1.2 Medical goods - Over the counter meds	5. Retailers and other providers of medical goods	391
1.2 Curative care - Day	1. Hospitals	388
2.1 Rehabilitative care - Inpatient	1. Hospitals	385
5.1.3 Medical goods - Other medical non-durables	5. Retailers and other providers of medical goods	379
2.3 Rehabilitative care - Outpatient	3. Providers of ambulatory care	374
1. Curative care	1.2 Hospitals - Mental health	371
1. Curative care	9. Rest of world	369
1.1 Curative care - Inpatient	1.3 Hospitals - Specialized	362
1.3.3 Curative care - Outpatient - Specialized	3.1 Providers of ambulatory care - Medical practices	360
3.1 Long-term care - Inpatient	1. Hospitals	360
1. Curative care	3.4 Providers of ambulatory care - Ambulatory health care centers	354
5. Medical goods	3. Providers of ambulatory care	351
2. Rehabilitative care	1.1 Hospitals - General	347
1.3.3 Curative care - Outpatient - Specialized	1. Hospitals	347
1.4 Curative care - Home-based	3. Providers of ambulatory care	347

1.2 Curative care - Day	1.1 Hospitals - General	341
4. Ancillary care	1.1 Hospitals - General	340
1.1 Curative care - Inpatient	3. Providers of ambulatory care	340
6. Preventative care	3.1 Providers of ambulatory care - Medical practices	338
6. Preventative care	3.4 Providers of ambulatory care - Ambulatory health care centers	335
1. Curative care	1.3 Hospitals - Specialized	331
6.1 Prevention care - IEC programs	HP Total	327
1.1 Curative care - Inpatient	9. Rest of world	325
1.3.3 Curative care - Outpatient - Specialized	1.1 Hospitals - General	321
5. Medical goods	1. Hospitals	318
HC Total	N.E.C.	317
1.3.1 Curative care - Outpatient - General	1. Hospitals	316
3. Long-term care	8. Rest of economy	314
1.3.nec Curative care - Outpatient - NEC	3. Providers of ambulatory care	313
3.2 Long-term care - Day	HP Total	299
2. Rehabilitative care	3.3 Providers of ambulatory care - Other practices	298
6.2 Prevention care - Immunization programs	HP Total	297
6.4 Prevention care - Healthy condition monitoring programs	HP Total	297
3. Long-term care	1.1 Hospitals - General	294
2.1 Rehabilitative care - Inpatient	1.1 Hospitals - General	292
3.1 Long-term care - Inpatient	1.1 Hospitals - General	292
2.3 Rehabilitative care - Outpatient	1. Hospitals	286
4. Ancillary care	8. Rest of economy	284
1.3.1 Curative care - Outpatient - General	1.1 Hospitals - General	284
6. Preventative care	7. Health care system admin.	282
6. Preventative care	1.1 Hospitals - General	281
3.4 Long-term care - Home-based	8. Rest of economy	281

2. Rehabilitative care	3.4 Providers of ambulatory care - Ambulatory health care centers	279
6.5 Prevention care - Epi surveillance and risk and disease control programs	HP Total	276
2. Rehabilitative care	1.3 Hospitals - Specialized	272
2.1 Rehabilitative care - Inpatient	1.3 Hospitals - Specialized	270
4. Ancillary care	3.1 Providers of ambulatory care - Medical practices	269
5.1 Medical goods - Pharms & other medical non-durable goods	1. Hospitals	268
2.3 Rehabilitative care - Outpatient	3.3 Providers of ambulatory care - Other practices	268
1.3.nec Curative care - Outpatient - NEC	3.3 Providers of ambulatory care - Other practices	265
1.2 Curative care - Day	3. Providers of ambulatory care	264
1.3 Curative care - Outpatient	1.2 Hospitals - Mental health	263
5.1 Medical goods - Pharms & other medical non-durable goods	3. Providers of ambulatory care	263
2. Rehabilitative care	2. Resid. long-term care fac.	261
1. Curative care	8. Rest of economy	258
1.1 Curative care - Inpatient	3.1 Providers of ambulatory care - Medical practices	255
6.1 Prevention care - IEC programs	6. Providers of preventative care	252
2.3 Rehabilitative care - Outpatient	3.4 Providers of ambulatory care - Ambulatory health care centers	252
6. Preventative care	4. Providers of ancillary services	249
1.3.1 Curative care - Outpatient - General	3.4 Providers of ambulatory care - Ambulatory health care centers	248
1.3 Curative care - Outpatient	1.3 Hospitals - Specialized	246
5. Medical goods	1.1 Hospitals - General	239
1. Curative care	2. Resid. long-term care fac.	238
6.3 Prevention care - Early disease detection	HP Total	236
2. Rehabilitative care	3.1 Providers of ambulatory care - Medical practices	232

1.3 Curative care - Outpatient	8. Rest of economy	231
2.2 Rehabilitative care - Day	HP Total	228
6. Preventative care	3.3 Providers of ambulatory care - Other practices	226
1.3 Curative care - Outpatient	9. Rest of world	226
1.3.2 Curative care - Outpatient - Dental	1. Hospitals	223
1.3.3 Curative care - Outpatient - Specialized	3.4 Providers of ambulatory care - Ambulatory health care centers	223
1.3.3 Curative care - Outpatient - Specialized	1.3 Hospitals - Specialized	223
5. Medical goods	3.1 Providers of ambulatory care - Medical practices	220
5.1 Medical goods - Pharms & other medical non-durable goods	1.1 Hospitals - General	218
9. Other health care services	N.E.C.	216
3. Long-term care	3.4 Providers of ambulatory care - Ambulatory health care centers	215
2.3 Rehabilitative care - Outpatient	3.1 Providers of ambulatory care - Medical practices	214
6.5 Prevention care - Epi surveillance and risk and disease control programs	6. Providers of preventative care	213
1.4 Curative care - Home-based	3.1 Providers of ambulatory care - Medical practices	213
1.4 Curative care - Home-based	1. Hospitals	211
1. Curative care	3.5 Providers of home health care services	207
2.2 Rehabilitative care - Day	1. Hospitals	206
4. Ancillary care	3.4 Providers of ambulatory care - Ambulatory health care centers	203
1.3 Curative care - Outpatient	2. Resid. long-term care fac.	199
3.1 Long-term care - Inpatient	1.2 Hospitals - Mental health	198
3.1 Long-term care - Inpatient	3. Providers of ambulatory care	198
1.3.1 Curative care - Outpatient - General	3.3 Providers of ambulatory care - Other practices	197
2.3 Rehabilitative care - Outpatient	1.3 Hospitals - Specialized	197
1.3.1 Curative care - Outpatient - General	8. Rest of economy	196

3. Long-term care	1.3 Hospitals - Specialized	195
3.4 Long-term care - Home-based	1. Hospitals	195
6. Preventative care	3.2 Providers of ambulatory care - Dental practices	193
4. Ancillary care	1.3 Hospitals - Specialized	193
1.3.3 Curative care - Outpatient - Specialized	1.2 Hospitals - Mental health	192
2.3 Rehabilitative care - Outpatient	1.1 Hospitals - General	190
6.4 Prevention care - Healthy condition monitoring programs	3. Providers of ambulatory care	190
2.1 Rehabilitative care - Inpatient	2. Resid. long-term care fac.	187
3. Long-term care	1.2 Hospitals - Mental health	186
3.4 Long-term care - Home-based	2. Resid. long-term care fac.	186
5. Medical goods	9. Rest of world	184
5.1.1 Medical goods - Prescribed meds	1. Hospitals	184
1.4 Curative care - Home-based	1.1 Hospitals - General	183
1.3.nec Curative care - Outpatient - NEC	1. Hospitals	181
1.3.nec Curative care - Outpatient - NEC	3.4 Providers of ambulatory care - Ambulatory health care centers	181
1.3.2 Curative care - Outpatient - Dental	1.1 Hospitals - General	180
2. Rehabilitative care	1.2 Hospitals - Mental health	177
3. Long-term care	3.1 Providers of ambulatory care - Medical practices	177
3.1 Long-term care - Inpatient	1.3 Hospitals - Specialized	175
1.3.3 Curative care - Outpatient - Specialized	3.3 Providers of ambulatory care - Other practices	174
3.2 Long-term care - Day	2. Resid. long-term care fac.	174
1.2 Curative care - Day	1.3 Hospitals - Specialized	174
3.4 Long-term care - Home-based	3.4 Providers of ambulatory care - Ambulatory health care centers	173
1.4 Curative care - Home-based	3.5 Providers of home health care services	172
1.2 Curative care - Day	3.1 Providers of ambulatory care - Medical practices	171

5.1 Medical goods - Pharms & other medical non-durable goods	3.1 Providers of ambulatory care - Medical practices	169
1.3.1 Curative care - Outpatient - General	9. Rest of world	168
6.4 Prevention care - Healthy condition monitoring programs	6. Providers of preventative care	168
1.3.2 Curative care - Outpatient - Dental	3.4 Providers of ambulatory care - Ambulatory health care centers	168
6.3 Prevention care - Early disease detection	6. Providers of preventative care	167
1. Curative care	5. Retailers and other providers of medical goods	166
5.1.1 Medical goods - Prescribed meds	1.1 Hospitals - General	166
3.3 Long-term care - Outpatient	HP Total	166
1.2 Curative care - Day	3.4 Providers of ambulatory care - Ambulatory health care centers	166
1.4 Curative care - Home-based	3.4 Providers of ambulatory care - Ambulatory health care centers	164
6.2 Prevention care - Immunization programs	6. Providers of preventative care	162
6. Preventative care	1.3 Hospitals - Specialized	162
6.1 Prevention care - IEC programs	3. Providers of ambulatory care	160
1.1 Curative care - Inpatient	3.4 Providers of ambulatory care - Ambulatory health care centers	160
2.1 Rehabilitative care - Inpatient	1.2 Hospitals - Mental health	158
1.3.nec Curative care - Outpatient - NEC	1.1 Hospitals - General	158
5.1 Medical goods - Pharms & other medical non-durable goods	9. Rest of world	158
2.2 Rehabilitative care - Day	1.1 Hospitals - General	157
5. Medical goods	8. Rest of economy	156
6.4 Prevention care - Healthy condition monitoring programs	1. Hospitals	156
5.2 Medical goods - Therapeutic appliances & prosthetics	3. Providers of ambulatory care	151
6.nec Prevention care - NEC	HP Total	150
1.3.1 Curative care - Outpatient - General	1.3 Hospitals - Specialized	150

5.2 Medical goods - Therapeutic appliances & prosthetics	1. Hospitals	147
1.2 Curative care - Day	1.2 Hospitals - Mental health	146
5. Medical goods	3.4 Providers of ambulatory care - Ambulatory health care centers	145
3.4 Long-term care - Home-based	1.1 Hospitals - General	145
7. Governance & admin	6. Providers of preventative care	143
7. Governance & admin	9. Rest of world	142
6.3 Prevention care - Early disease detection	3. Providers of ambulatory care	142
3.2 Long-term care - Day	1. Hospitals	142
6.4 Prevention care - Healthy condition monitoring programs	3.1 Providers of ambulatory care - Medical practices	142
1. Curative care	6. Providers of preventative care	140
2.4 Rehabilitative care - Home-based	HP Total	139
6.3 Prevention care - Early disease detection	1. Hospitals	137
5.2 Medical goods - Therapeutic appliances & prosthetics	1.1 Hospitals - General	136
5.1.1 Medical goods - Prescribed meds	3. Providers of ambulatory care	135
6.4 Prevention care - Healthy condition monitoring programs	1.1 Hospitals - General	133
2.1 Rehabilitative care - Inpatient	3. Providers of ambulatory care	129
6.6 Prevention care - Preparing for disaster and emergency response programs	HP Total	127
6.4 Prevention care - Healthy condition monitoring programs	8. Rest of economy	127
1.3.1 Curative care - Outpatient - General	3.2 Providers of ambulatory care - Dental practices	126
3. Long-term care	3.3 Providers of ambulatory care - Other practices	126
6. Preventative care	2. Resid. long-term care fac.	125
3.4 Long-term care - Home-based	3.1 Providers of ambulatory care - Medical practices	125
5.1 Medical goods - Pharms & other medical non-durable goods	3.4 Providers of ambulatory care - Ambulatory health care centers	124

1.3.nec Curative care - Outpatient - NEC	3.1 Providers of ambulatory care - Medical practices	123
1.1 Curative care - Inpatient	2. Resid. long-term care fac.	123
2.2 Rehabilitative care - Day	3. Providers of ambulatory care	122
6.2 Prevention care - Immunization programs	3. Providers of ambulatory care	121
4. Ancillary care	9. Rest of world	120
1. Curative care	4. Providers of ancillary services	120
5.1.1 Medical goods - Prescribed meds	3.1 Providers of ambulatory care - Medical practices	120
1.3.1 Curative care - Outpatient - General	1.2 Hospitals - Mental health	117
1.3.2 Curative care - Outpatient - Dental	3.1 Providers of ambulatory care - Medical practices	117
2.3 Rehabilitative care - Outpatient	2. Resid. long-term care fac.	116
1.3 Curative care - Outpatient	6. Providers of preventative care	115
6. Preventative care	9. Rest of world	115
4. Ancillary care	1.2 Hospitals - Mental health	114
1.3.3 Curative care - Outpatient - Specialized	2. Resid. long-term care fac.	114
1.3 Curative care - Outpatient	5. Retailers and other providers of medical goods	113
5. Medical goods	7. Health care system admin.	112
3.1 Long-term care - Inpatient	3.4 Providers of ambulatory care - Ambulatory health care centers	112
5. Medical goods	3.2 Providers of ambulatory care - Dental practices	110
1.3.2 Curative care - Outpatient - Dental	9. Rest of world	110
6.3 Prevention care - Early disease detection	3.1 Providers of ambulatory care - Medical practices	110
1.3 Curative care - Outpatient	4. Providers of ancillary services	110
5.1 Medical goods - Pharms & other medical non-durable goods	8. Rest of economy	110
1.3 Curative care - Outpatient	7. Health care system admin.	109
1. Curative care	7. Health care system admin.	108

6.1 Prevention care - IEC programs	3.4 Providers of ambulatory care - Ambulatory health care centers	108
1.3.2 Curative care - Outpatient - Dental	8. Rest of economy	107
6.1 Prevention care - IEC programs	1.1 Hospitals - General	107
6. Preventative care	1.2 Hospitals - Mental health	107
2. Rehabilitative care	8. Rest of economy	106
6.5 Prevention care - Epi surveillance and risk and disease control programs	7. Health care system admin.	106
3.2 Long-term care - Day	3. Providers of ambulatory care	106
6.nec Prevention care - NEC	6. Providers of preventative care	105
5.2 Medical goods - Therapeutic appliances & prosthetics	8. Rest of economy	105
6.1 Prevention care - IEC programs	8. Rest of economy	104
6.3 Prevention care - Early disease detection	1.1 Hospitals - General	102
1.1 Curative care - Inpatient	3.3 Providers of ambulatory care - Other practices	99
7. Governance & admin	8. Rest of economy	99
1.1 Curative care - Inpatient	8. Rest of economy	98
1.3.1 Curative care - Outpatient - General	7. Health care system admin.	97
1.3.1 Curative care - Outpatient - General	2. Resid. long-term care fac.	97
3.4 Long-term care - Home-based	1.3 Hospitals - Specialized	97
3.4 Long-term care - Home-based	3.3 Providers of ambulatory care - Other practices	96
6.1 Prevention care - IEC programs	1. Hospitals	95
1.3.2 Curative care - Outpatient - Dental	1.3 Hospitals - Specialized	95
1.3.3 Curative care - Outpatient - Specialized	9. Rest of world	95
6.4 Prevention care - Healthy condition monitoring programs	3.4 Providers of ambulatory care - Ambulatory health care centers	95
1.3.3 Curative care - Outpatient - Specialized	8. Rest of economy	93
1.3.3 Curative care - Outpatient - Specialized	3.2 Providers of ambulatory care - Dental practices	91
9. Other health care services	3. Providers of ambulatory care	91

6.4 Prevention care - Healthy condition monitoring programs	3.2 Providers of ambulatory care - Dental practices	89
5.2 Medical goods - Therapeutic appliances & prosthetics	3.4 Providers of ambulatory care - Ambulatory health care centers	89
2.4 Rehabilitative care - Home-based	1. Hospitals	88
2.4 Rehabilitative care - Home-based	3. Providers of ambulatory care	88
1.4 Curative care - Home-based	3.3 Providers of ambulatory care - Other practices	87
4. Ancillary care	6. Providers of preventative care	86
5.1.3 Medical goods - Other medical non-durables	3. Providers of ambulatory care	86
3.1 Long-term care - Inpatient	8. Rest of economy	86
2.1 Rehabilitative care - Inpatient	3.4 Providers of ambulatory care - Ambulatory health care centers	85
6. Preventative care	5. Retailers and other providers of medical goods	85
3.3 Long-term care - Outpatient	3. Providers of ambulatory care	85
6.4 Prevention care - Healthy condition monitoring programs	7. Health care system admin.	85
3.1 Long-term care - Inpatient	3.1 Providers of ambulatory care - Medical practices	85
1.3.nec Curative care - Outpatient - NEC	1.3 Hospitals - Specialized	85
5. Medical goods	6. Providers of preventative care	84
5.1.1 Medical goods - Prescribed meds	9. Rest of world	84
6.6 Prevention care - Preparing for disaster and emergency response programs	6. Providers of preventative care	81
5.1 Medical goods - Pharms & other medical non-durable goods	7. Health care system admin.	81
2.2 Rehabilitative care - Day	1.3 Hospitals - Specialized	81
5. Medical goods	4. Providers of ancillary services	81
5.1 Medical goods - Pharms & other medical non-durable goods	3.2 Providers of ambulatory care - Dental practices	81
2.3 Rehabilitative care - Outpatient	8. Rest of economy	81
9. Other health care services	8. Rest of economy	80

4. Ancillary care	3.2 Providers of ambulatory care - Dental practices	80
5. Medical goods	1.3 Hospitals - Specialized	80
6.2 Prevention care - Immunization programs	3.1 Providers of ambulatory care - Medical practices	80
5.1 Medical goods - Pharms & other medical non-durable goods	1.2 Hospitals - Mental health	78
6. Preventative care	3.5 Providers of home health care services	77
5.1.1 Medical goods - Prescribed meds	3.4 Providers of ambulatory care - Ambulatory health care centers	76
3.3 Long-term care - Outpatient	1. Hospitals	76
5. Medical goods	1.2 Hospitals - Mental health	75
6.1 Prevention care - IEC programs	7. Health care system admin.	75
1.3 Curative care - Outpatient	3.5 Providers of home health care services	75
6.1 Prevention care - IEC programs	3.3 Providers of ambulatory care - Other practices	75
2.3 Rehabilitative care - Outpatient	1.2 Hospitals - Mental health	75
9. Other health care services	7. Health care system admin.	74
1.3.1 Curative care - Outpatient - General	6. Providers of preventative care	74
9. Other health care services	1. Hospitals	73
5.2 Medical goods - Therapeutic appliances & prosthetics	3.1 Providers of ambulatory care - Medical practices	73
1.4 Curative care - Home-based	8. Rest of economy	73
5.1 Medical goods - Pharms & other medical non-durable goods	6. Providers of preventative care	72
1.3.nec Curative care - Outpatient - NEC	9. Rest of world	72
2.2 Rehabilitative care - Day	3.4 Providers of ambulatory care - Ambulatory health care centers	72
5.1 Medical goods - Pharms & other medical non-durable goods	4. Providers of ancillary services	72
6.1 Prevention care - IEC programs	3.1 Providers of ambulatory care - Medical practices	72
1. nec Curative care - NEC	HP Total	72
1.1 Curative care - Inpatient	6. Providers of preventative care	71

2.2 Rehabilitative care - Day	3.1 Providers of ambulatory care - Medical practices	70
2. Rehabilitative care	3.5 Providers of home health care services	70
1.3.nec Curative care - Outpatient - NEC	8. Rest of economy	69
6.3 Prevention care - Early disease detection	3.4 Providers of ambulatory care - Ambulatory health care centers	69
6.4 Prevention care - Healthy condition monitoring programs	1.3 Hospitals - Specialized	69
5. Medical goods	2. Resid. long-term care fac.	69
7. Governance & admin	3. Providers of ambulatory care	68
HC Total	1.nec Hospitals - Other	68
1.3.nec Curative care - Outpatient - NEC	1.2 Hospitals - Mental health	67
5. Medical goods	3.3 Providers of ambulatory care - Other practices	67
1.4 Curative care - Home-based	1.3 Hospitals - Specialized	67
3.2 Long-term care - Day	1.1 Hospitals - General	66
1.3.1 Curative care - Outpatient - General	4. Providers of ancillary services	66
5.1.1 Medical goods - Prescribed meds	3.2 Providers of ambulatory care - Dental practices	66
5.1.1 Medical goods - Prescribed meds	1.2 Hospitals - Mental health	63
5.2 Medical goods - Therapeutic appliances & prosthetics	9. Rest of world	63
9. Other health care services	9. Rest of world	63
1.4 Curative care - Home-based	2. Resid. long-term care fac.	63
3.2 Long-term care - Day	1.2 Hospitals - Mental health	63
5.1.1 Medical goods - Prescribed meds	1.3 Hospitals - Specialized	63
5.2 Medical goods - Therapeutic appliances & prosthetics	3.2 Providers of ambulatory care - Dental practices	62
3. Long-term care	7. Health care system admin.	62
2. Rehabilitative care	9. Rest of world	62
5.1 Medical goods - Pharms & other medical non-durable goods	1.3 Hospitals - Specialized	61
5.1.1 Medical goods - Prescribed meds	7. Health care system admin.	61

6.1 Prevention care - IEC programs	1.3 Hospitals - Specialized	60
5.2 Medical goods - Therapeutic appliances & prosthetics	1.3 Hospitals - Specialized	60
1.3.nec Curative care - Outpatient - NEC	2. Resid. long-term care fac.	60
6.2 Prevention care - Immunization programs	3.4 Providers of ambulatory care - Ambulatory health care centers	59
1.3.3 Curative care - Outpatient - Specialized	4. Providers of ancillary services	59
4. Ancillary care	3.3 Providers of ambulatory care - Other practices	59
1.3.2 Curative care - Outpatient - Dental	2. Resid. long-term care fac.	59
5.1.3 Medical goods - Other medical non-durables	1.1 Hospitals - General	59
2.2 Rehabilitative care - Day	1.2 Hospitals - Mental health	58
2. Rehabilitative care	4. Providers of ancillary services	58
3.1 Long-term care - Inpatient	3.5 Providers of home health care services	58
5.1.3 Medical goods - Other medical non-durables	1. Hospitals	58
2.1 Rehabilitative care - Inpatient	8. Rest of economy	58
4. Ancillary care	5. Retailers and other providers of medical goods	57
5.2 Medical goods - Therapeutic appliances & prosthetics	3.3 Providers of ambulatory care - Other practices	57
2.1 Rehabilitative care - Inpatient	3.1 Providers of ambulatory care - Medical practices	57
1.4 Curative care - Home-based	3.2 Providers of ambulatory care - Dental practices	57
3.2 Long-term care - Day	1.3 Hospitals - Specialized	56
3. Long-term care	5. Retailers and other providers of medical goods	56
1.4 Curative care - Home-based	1.2 Hospitals - Mental health	56
1.2 Curative care - Day	2. Resid. long-term care fac.	56
2.4 Rehabilitative care - Home-based	1.1 Hospitals - General	56
7. Governance & admin	1. Hospitals	55
9. Other health care services	1.1 Hospitals - General	55
4. Ancillary care	2. Resid. long-term care fac.	55

2. Rehabilitative care	7. Health care system admin.	55
1.3.3 Curative care - Outpatient - Specialized	5. Retailers and other providers of medical goods	55
1.3 Curative care - Outpatient	N.E.C.	55
4. Ancillary care	7. Health care system admin.	54
9. Other health care services	6. Providers of preventative care	54
1.1 Curative care - Inpatient	N.E.C.	54
1.3.2 Curative care - Outpatient - Dental	3.3 Providers of ambulatory care - Other practices	54
1.3.2 Curative care - Outpatient - Dental	5. Retailers and other providers of medical goods	54
1.1 Curative care - Inpatient	3.2 Providers of ambulatory care - Dental practices	53
3.2 Long-term care - Day	3.4 Providers of ambulatory care - Ambulatory health care centers	51
6.5 Prevention care - Epi surveillance and risk and disease control programs	3. Providers of ambulatory care	51
6.4 Prevention care - Healthy condition monitoring programs	3.3 Providers of ambulatory care - Other practices	51
5.1 Medical goods - Pharms & other medical non-durable goods	3.3 Providers of ambulatory care - Other practices	50
2.1 Rehabilitative care - Inpatient	9. Rest of world	50
3. Long-term care	9. Rest of world	50
3.4 Long-term care - Home-based	1.2 Hospitals - Mental health	50
1.1 Curative care - Inpatient	7. Health care system admin.	49
1.3.1 Curative care - Outpatient - General	3.5 Providers of home health care services	49
5.1.1 Medical goods - Prescribed meds	6. Providers of preventative care	48
6.5 Prevention care - Epi surveillance and risk and disease control programs	8. Rest of economy	48
1. Curative care	N.E.C.	47
3.1 Long-term care - Inpatient	7. Health care system admin.	47
3.4 Long-term care - Home-based	7. Health care system admin.	47
5.1.3 Medical goods - Other medical non-durables	3.4 Providers of ambulatory care - Ambulatory health care centers	47

9. Other health care services	3.3 Providers of ambulatory care - Other practices	47
1.3.nec Curative care - Outpatient - NEC	4. Providers of ancillary services	47
1.1 Curative care - Inpatient	4. Providers of ancillary services	46
6.3 Prevention care - Early disease detection	1.3 Hospitals - Specialized	46
3.3 Long-term care - Outpatient	1.2 Hospitals - Mental health	46
2. Rehabilitative care	5. Retailers and other providers of medical goods	46
5.2 Medical goods - Therapeutic appliances & prosthetics	2. Resid. long-term care fac.	45
1.2 Curative care - Day	8. Rest of economy	45
1.2 Curative care - Day	9. Rest of world	44
6. Preventative care	N.E.C.	44
2.4 Rehabilitative care - Home-based	3.5 Providers of home health care services	43
5.2 Medical goods - Therapeutic appliances & prosthetics	4. Providers of ancillary services	43
2. Rehabilitative care	6. Providers of preventative care	42
1.3.2 Curative care - Outpatient - Dental	7. Health care system admin.	42
6.3 Prevention care - Early disease detection	7. Health care system admin.	41
6.2 Prevention care - Immunization programs	1. Hospitals	41
5.1.2 Medical goods - Over the counter meds	3. Providers of ambulatory care	41
1.3.nec Curative care - Outpatient - NEC	5. Retailers and other providers of medical goods	41
7. Governance & admin	3.4 Providers of ambulatory care - Ambulatory health care centers	40
1.3.3 Curative care - Outpatient - Specialized	7. Health care system admin.	40
6.3 Prevention care - Early disease detection	8. Rest of economy	40
6.5 Prevention care - Epi surveillance and risk and disease control programs	1. Hospitals	39
9. Other health care services	3.4 Providers of ambulatory care - Ambulatory health care centers	39
6.2 Prevention care - Immunization programs	1.1 Hospitals - General	39

5.1.3 Medical goods - Other medical non-durables	3.3 Providers of ambulatory care - Other practices	39
1.3.3 Curative care - Outpatient - Specialized	3.5 Providers of home health care services	39
2.4 Rehabilitative care - Home-based	3.3 Providers of ambulatory care - Other practices	39
3.3 Long-term care - Outpatient	1.1 Hospitals - General	39
2. Rehabilitative care	3.2 Providers of ambulatory care - Dental practices	39
5. Medical goods	3.5 Providers of home health care services	39
5.1 Medical goods - Pharms & other medical non-durable goods	2. Resid. long-term care fac.	39
3.4 Long-term care - Home-based	5. Retailers and other providers of medical goods	38
9. Other health care services	2. Resid. long-term care fac.	38
5.1.3 Medical goods - Other medical non-durables	8. Rest of economy	38
1.4 Curative care - Home-based	5. Retailers and other providers of medical goods	38
3.2 Long-term care - Day	3.5 Providers of home health care services	38
6.3 Prevention care - Early disease detection	4. Providers of ancillary services	37
1.3.1 Curative care - Outpatient - General	5. Retailers and other providers of medical goods	37
2.4 Rehabilitative care - Home-based	3.4 Providers of ambulatory care - Ambulatory health care centers	36
3.1 Long-term care - Inpatient	3.3 Providers of ambulatory care - Other practices	36
9. Other health care services	5. Retailers and other providers of medical goods	36
7. Governance & admin	1.1 Hospitals - General	35
6.nec Prevention care - NEC	3. Providers of ambulatory care	35
5.1 Medical goods - Pharms & other medical non-durable goods	3.5 Providers of home health care services	35
3.1 Long-term care - Inpatient	9. Rest of world	35
1.1 Curative care - Inpatient	5. Retailers and other providers of medical goods	34
6.2 Prevention care - Immunization programs	7. Health care system admin.	34

3.nec Long-term care - NEC	HP Total	34
3.3 Long-term care - Outpatient	3.1 Providers of ambulatory care - Medical practices	34
6.6 Prevention care - Preparing for disaster and emergency response programs	7. Health care system admin.	34
3.3 Long-term care - Outpatient	3.3 Providers of ambulatory care - Other practices	34
5.1.3 Medical goods - Other medical non-durables	3.5 Providers of home health care services	34
3.2 Long-term care - Day	3.1 Providers of ambulatory care - Medical practices	34
2.4 Rehabilitative care - Home-based	3.1 Providers of ambulatory care - Medical practices	34
5.1.3 Medical goods - Other medical non-durables	4. Providers of ancillary services	33
3.3 Long-term care - Outpatient	2. Resid. long-term care fac.	33
6.4 Prevention care - Healthy condition monitoring programs	4. Providers of ancillary services	33
9. Other health care services	1.3 Hospitals - Specialized	33
2.3 Rehabilitative care - Outpatient	4. Providers of ancillary services	33
6.1 Prevention care - IEC programs	9. Rest of world	32
5. Medical goods	N.E.C.	32
5.2 Medical goods - Therapeutic appliances & prosthetics	7. Health care system admin.	32
3.3 Long-term care - Outpatient	3.4 Providers of ambulatory care - Ambulatory health care centers	32
6.2 Prevention care - Immunization programs	8. Rest of economy	32
1.3.3 Curative care - Outpatient - Specialized	N.E.C.	32
6.nec Prevention care - NEC	1.1 Hospitals - General	31
1.3.3 Curative care - Outpatient - Specialized	6. Providers of preventative care	31
6.nec Prevention care - NEC	1. Hospitals	31
6.nec Prevention care - NEC	3.4 Providers of ambulatory care - Ambulatory health care centers	31
3. Long-term care	4. Providers of ancillary services	31
5.1.1 Medical goods - Prescribed meds	4. Providers of ancillary services	31

1.3.1 Curative care - Outpatient - General	N.E.C.	31
1.2 Curative care - Day	3.2 Providers of ambulatory care - Dental practices	31
5.1.2 Medical goods - Over the counter meds	3.1 Providers of ambulatory care - Medical practices	31
4. Ancillary care	N.E.C.	30
7. Governance & admin	3.1 Providers of ambulatory care - Medical practices	30
3.4 Long-term care - Home-based	4. Providers of ancillary services	30
2.3 Rehabilitative care - Outpatient	3.5 Providers of home health care services	30
5.1.3 Medical goods - Other medical non-durables	1.3 Hospitals - Specialized	30
5.2 Medical goods - Therapeutic appliances & prosthetics	3.5 Providers of home health care services	30
5.2 Medical goods - Therapeutic appliances & prosthetics	6. Providers of preventative care	29
5.1.3 Medical goods - Other medical non-durables	7. Health care system admin.	29
6.5 Prevention care - Epi surveillance and risk and disease control programs	3.4 Providers of ambulatory care - Ambulatory health care centers	29
2.nec Rehabilitative care - NEC	HP Total	29
9. Other health care services	3.1 Providers of ambulatory care - Medical practices	29
9. Other health care services	4. Providers of ancillary services	29
1.3.nec Curative care - Outpatient - NEC	3.2 Providers of ambulatory care - Dental practices	29
5.1.3 Medical goods - Other medical non-durables	3.1 Providers of ambulatory care - Medical practices	29
2.2 Rehabilitative care - Day	2. Resid. long-term care fac.	29
4. Ancillary care	3.5 Providers of home health care services	28
5.nec Medical goods - NEC	HP Total	28
6.nec Prevention care - NEC	7. Health care system admin.	28
6.5 Prevention care - Epi surveillance and risk and disease control programs	1.1 Hospitals - General	28
6.1 Prevention care - IEC programs	4. Providers of ancillary services	28
6.nec Prevention care - NEC	8. Rest of economy	28

5.1.2 Medical goods - Over the counter meds	1. Hospitals	28
2.1 Rehabilitative care - Inpatient	7. Health care system admin.	27
7. Governance & admin	4. Providers of ancillary services	27
3.3 Long-term care - Outpatient	1.3 Hospitals - Specialized	27
6.3 Prevention care - Early disease detection	3.2 Providers of ambulatory care - Dental practices	26
5.1.2 Medical goods - Over the counter meds	8. Rest of economy	26
2.3 Rehabilitative care - Outpatient	5. Retailers and other providers of medical goods	26
1. nec Curative care - NEC	1.1 Hospitals - General	26
5.1.3 Medical goods - Other medical non-durables	9. Rest of world	26
6.1 Prevention care - IEC programs	3.2 Providers of ambulatory care - Dental practices	26
2.3 Rehabilitative care - Outpatient	6. Providers of preventative care	25
5.1 Medical goods - Pharms & other medical non-durable goods	N.E.C.	25
6.4 Prevention care - Healthy condition monitoring programs	1.2 Hospitals - Mental health	25
1.3.nec Curative care - Outpatient - NEC	3.5 Providers of home health care services	25
3.2 Long-term care - Day	8. Rest of economy	25
1.3.nec Curative care - Outpatient - NEC	6. Providers of preventative care	24
6.2 Prevention care - Immunization programs	5. Retailers and other providers of medical goods	24
2.4 Rehabilitative care - Home-based	2. Resid. long-term care fac.	24
1.3 Curative care - Outpatient	1.nec Hospitals - Other	24
HC Total	3.nec Providers of ambulatory care - NEC	24
7. Governance & admin	N.E.C.	24
6.1 Prevention care - IEC programs	5. Retailers and other providers of medical goods	24
2.4 Rehabilitative care - Home-based	1.3 Hospitals - Specialized	23
1.3.2 Curative care - Outpatient - Dental	1.2 Hospitals - Mental health	23
3. Long-term care	3.2 Providers of ambulatory care - Dental practices	23

3.1 Long-term care - Inpatient	5. Retailers and other providers of medical goods	23
6.5 Prevention care - Epi surveillance and risk and disease control programs	4. Providers of ancillary services	22
6.1 Prevention care - IEC programs	1.2 Hospitals - Mental health	22
1.1 Curative care - Inpatient	1.nec Hospitals - Other	22
5.1.1 Medical goods - Prescribed meds	N.E.C.	22
6.5 Prevention care - Epi surveillance and risk and disease control programs	1.3 Hospitals - Specialized	21
6.6 Prevention care - Preparing for disaster and emergency response programs	1. Hospitals	21
6.6 Prevention care - Preparing for disaster and emergency response programs	1.1 Hospitals - General	21
2.1 Rehabilitative care - Inpatient	5. Retailers and other providers of medical goods	21
3.4 Long-term care - Home-based	3.2 Providers of ambulatory care - Dental practices	21
5.1.1 Medical goods - Prescribed meds	3.5 Providers of home health care services	21
2.2 Rehabilitative care - Day	8. Rest of economy	21
2.3 Rehabilitative care - Outpatient	9. Rest of world	21
2.4 Rehabilitative care - Home-based	3.2 Providers of ambulatory care - Dental practices	21
5.2 Medical goods - Therapeutic appliances & prosthetics	N.E.C.	20
5.1.2 Medical goods - Over the counter meds	6. Providers of preventative care	20
3.3 Long-term care - Outpatient	3.5 Providers of home health care services	20
2.2 Rehabilitative care - Day	3.3 Providers of ambulatory care - Other practices	20
2.2 Rehabilitative care - Day	4. Providers of ancillary services	20
9. Other health care services	1.2 Hospitals - Mental health	20
1.2 Curative care - Day	5. Retailers and other providers of medical goods	20
1.3.2 Curative care - Outpatient - Dental	N.E.C.	20
5.1.1 Medical goods - Prescribed meds	8. Rest of economy	20

5.1.3 Medical goods - Other medical non-durables	3.2 Providers of ambulatory care - Dental practices	20
1.4 Curative care - Home-based	9. Rest of world	20
3.3 Long-term care - Outpatient	8. Rest of economy	19
2. Rehabilitative care	N.E.C.	19
1.3.2 Curative care - Outpatient - Dental	4. Providers of ancillary services	19
5.1.3 Medical goods - Other medical non-durables	N.E.C.	19
2.3 Rehabilitative care - Outpatient	3.2 Providers of ambulatory care - Dental practices	19
5.1.3 Medical goods - Other medical non-durables	1.2 Hospitals - Mental health	19
2.4 Rehabilitative care - Home-based	1.2 Hospitals - Mental health	19
6.4 Prevention care - Healthy condition monitoring programs	3.5 Providers of home health care services	18
1.3.2 Curative care - Outpatient - Dental	3.5 Providers of home health care services	18
9. Other health care services	3.2 Providers of ambulatory care - Dental practices	18
6.4 Prevention care - Healthy condition monitoring programs	5. Retailers and other providers of medical goods	18
2.4 Rehabilitative care - Home-based	5. Retailers and other providers of medical goods	18
7. Governance & admin	5. Retailers and other providers of medical goods	17
1. nec Curative care - NEC	1. Hospitals	17
1.2 Curative care - Day	3.3 Providers of ambulatory care - Other practices	17
1.1 Curative care - Inpatient	3.5 Providers of home health care services	17
5.2 Medical goods - Therapeutic appliances & prosthetics	1.2 Hospitals - Mental health	17
1.2 Curative care - Day	N.E.C.	17
1.2 Curative care - Day	6. Providers of preventative care	17
5.1.3 Medical goods - Other medical non-durables	2. Resid. long-term care fac.	17
5.1.2 Medical goods - Over the counter meds	3.2 Providers of ambulatory care - Dental practices	17

5.1.1 Medical goods - Prescribed meds	3.3 Providers of ambulatory care - Other practices	16
2.4 Rehabilitative care - Home-based	4. Providers of ancillary services	16
6.1 Prevention care - IEC programs	3.5 Providers of home health care services	16
6.5 Prevention care - Epi surveillance and risk and disease control programs	3.1 Providers of ambulatory care - Medical practices	16
5.1.2 Medical goods - Over the counter meds	9. Rest of world	16
1.2 Curative care - Day	4. Providers of ancillary services	16
3.4 Long-term care - Home-based	9. Rest of world	16
6.nec Prevention care - NEC	9. Rest of world	15
1.4 Curative care - Home-based	4. Providers of ancillary services	15
6.6 Prevention care - Preparing for disaster and emergency response programs	4. Providers of ancillary services	15
6.3 Prevention care - Early disease detection	3.3 Providers of ambulatory care - Other practices	15
6.2 Prevention care - Immunization programs	1.3 Hospitals - Specialized	15
1.2 Curative care - Day	3.5 Providers of home health care services	15
9. Other health care services	3.5 Providers of home health care services	15
5.1.1 Medical goods - Prescribed meds	2. Resid. long-term care fac.	15
1.4 Curative care - Home-based	N.E.C.	15
2.3 Rehabilitative care - Outpatient	7. Health care system admin.	15
7. Governance & admin	1.3 Hospitals - Specialized	15
3. Long-term care	6. Providers of preventative care	14
2.1 Rehabilitative care - Inpatient	3.3 Providers of ambulatory care - Other practices	14
1.3.nec Curative care - Outpatient - NEC	7. Health care system admin.	14
5.1.2 Medical goods - Over the counter meds	3.4 Providers of ambulatory care - Ambulatory health care centers	13
2.1 Rehabilitative care - Inpatient	4. Providers of ancillary services	13
2.1 Rehabilitative care - Inpatient	N.E.C.	13
2.3 Rehabilitative care - Outpatient	N.E.C.	13
6.6 Prevention care - Preparing for disaster and emergency response programs	8. Rest of economy	13

6.nec Prevention care - NEC	N.E.C.	12
5.1.3 Medical goods - Other medical non-durables	6. Providers of preventative care	12
6.5 Prevention care - Epi surveillance and risk and disease control programs	9. Rest of world	12
1. Curative care	1.nec Hospitals - Other	12
3.1 Long-term care - Inpatient	4. Providers of ancillary services	12
3. Long-term care	N.E.C.	12
3.1 Long-term care - Inpatient	N.E.C.	12
3.4 Long-term care - Home-based	N.E.C.	12
6.nec Prevention care - NEC	5. Retailers and other providers of medical goods	12
6. Preventative care	1.nec Hospitals - Other	12
7. Governance & admin	1.2 Hospitals - Mental health	11
2.1 Rehabilitative care - Inpatient	3.5 Providers of home health care services	11
6.nec Prevention care - NEC	3.1 Providers of ambulatory care - Medical practices	11
6.3 Prevention care - Early disease detection	1.2 Hospitals - Mental health	11
6.6 Prevention care - Preparing for disaster and emergency response programs	1.2 Hospitals - Mental health	11
6.4 Prevention care - Healthy condition monitoring programs	2. Resid. long-term care fac.	11
2.nec Rehabilitative care - NEC	1. Hospitals	10
6.4 Prevention care - Healthy condition monitoring programs	9. Rest of world	10
2.1 Rehabilitative care - Inpatient	3.2 Providers of ambulatory care - Dental practices	10
1.3.nec Curative care - Outpatient - NEC	N.E.C.	10
6.nec Prevention care - NEC	3.3 Providers of ambulatory care - Other practices	9
5.nec Medical goods - NEC	5. Retailers and other providers of medical goods	9
6.6 Prevention care - Preparing for disaster and emergency response programs	1.3 Hospitals - Specialized	9

3.2 Long-term care - Day	3.3 Providers of ambulatory care - Other practices	9
6.nec Prevention care - NEC	1.3 Hospitals - Specialized	9
6.1 Prevention care - IEC programs	2. Resid. long-term care fac.	9
6.nec Prevention care - NEC	4. Providers of ancillary services	9
6.nec Prevention care - NEC	1.2 Hospitals - Mental health	9
6.nec Prevention care - NEC	2. Resid. long-term care fac.	9
6.1 Prevention care - IEC programs	N.E.C.	8
2.4 Rehabilitative care - Home-based	8. Rest of economy	8
1.2 Curative care - Day	7. Health care system admin.	8
3.3 Long-term care - Outpatient	7. Health care system admin.	8
6.3 Prevention care - Early disease detection	2. Resid. long-term care fac.	8
6.3 Prevention care - Early disease detection	5. Retailers and other providers of medical goods	8
1.3 Curative care - Outpatient	3.nec Providers of ambulatory care - NEC	8
1. nec Curative care - NEC	1.3 Hospitals - Specialized	8
6.2 Prevention care - Immunization programs	3.3 Providers of ambulatory care - Other practices	8
6. Preventative care	3.nec Providers of ambulatory care - NEC	8
7. Governance & admin	2. Resid. long-term care fac.	8
6.2 Prevention care - Immunization programs	1.2 Hospitals - Mental health	8
6.nec Prevention care - NEC	1.nec Hospitals - Other	8
2.2 Rehabilitative care - Day	7. Health care system admin.	7
3.2 Long-term care - Day	7. Health care system admin.	7
3.4 Long-term care - Home-based	6. Providers of preventative care	7
5.1.2 Medical goods - Over the counter meds	N.E.C.	7
6.3 Prevention care - Early disease detection	3.5 Providers of home health care services	7
1. nec Curative care - NEC	N.E.C.	7
4. Ancillary care	1.nec Hospitals - Other	7
4. Ancillary care	3.nec Providers of ambulatory care - NEC	7
1.1 Curative care - Inpatient	3.nec Providers of ambulatory care - NEC	7

2.nec Rehabilitative care - NEC	1.1 Hospitals - General	7
2.4 Rehabilitative care - Home-based	6. Providers of preventative care	7
5.1.2 Medical goods - Over the counter meds	1.1 Hospitals - General	6
1.3.2 Curative care - Outpatient - Dental	6. Providers of preventative care	6
1. nec Curative care - NEC	1.nec Hospitals - Other	6
6.2 Prevention care - Immunization programs	9. Rest of world	6
6.2 Prevention care - Immunization programs	N.E.C.	6
6.3 Prevention care - Early disease detection	N.E.C.	6
6.4 Prevention care - Healthy condition monitoring programs	N.E.C.	6
6.6 Prevention care - Preparing for disaster and emergency response programs	3. Providers of ambulatory care	6
1. Curative care	3.nec Providers of ambulatory care - NEC	6
6.6 Prevention care - Preparing for disaster and emergency response programs	3.4 Providers of ambulatory care - Ambulatory health care centers	6
5.1.2 Medical goods - Over the counter meds	7. Health care system admin.	6
6.2 Prevention care - Immunization programs	4. Providers of ancillary services	5
1.3.1 Curative care - Outpatient - General	1.nec Hospitals - Other	5
1.3.nec Curative care - Outpatient - NEC	1.nec Hospitals - Other	5
3.nec Long-term care - NEC	8. Rest of economy	5
6.6 Prevention care - Preparing for disaster and emergency response programs	9. Rest of world	5
2.2 Rehabilitative care - Day	3.5 Providers of home health care services	5
5. Medical goods	1.nec Hospitals - Other	5
5.1 Medical goods - Pharms & other medical non-durable goods	1.nec Hospitals - Other	5
1. nec Curative care - NEC	3. Providers of ambulatory care	5
1. nec Curative care - NEC	3.1 Providers of ambulatory care - Medical practices	5
1.4 Curative care - Home-based	6. Providers of preventative care	4
1.3.1 Curative care - Outpatient - General	3.nec Providers of ambulatory care - NEC	4
9. Other health care services	3.nec Providers of ambulatory care - NEC	4

9. Other health care services	1.nec Hospitals - Other	4
6.5 Prevention care - Epi surveillance and risk and disease control programs	3.3 Providers of ambulatory care - Other practices	4
5. Medical goods	3.nec Providers of ambulatory care - NEC	4
5.1.2 Medical goods - Over the counter meds	2. Resid. long-term care fac.	4
6.2 Prevention care - Immunization programs	2. Resid. long-term care fac.	4
1. nec Curative care - NEC	3.nec Providers of ambulatory care - NEC	4
6.3 Prevention care - Early disease detection	1.nec Hospitals - Other	4
6.2 Prevention care - Immunization programs	3.5 Providers of home health care services	4
5.1.2 Medical goods - Over the counter meds	1.2 Hospitals - Mental health	4
5.1.2 Medical goods - Over the counter meds	3.5 Providers of home health care services	4
6.6 Prevention care - Preparing for disaster and emergency response programs	3.5 Providers of home health care services	4
6.nec Prevention care - NEC	3.5 Providers of home health care services	4
3.2 Long-term care - Day	6. Providers of preventative care	3
1. nec Curative care - NEC	8. Rest of economy	3
2.nec Rehabilitative care - NEC	9. Rest of world	3
1. nec Curative care - NEC	9. Rest of world	3
2.1 Rehabilitative care - Inpatient	6. Providers of preventative care	3
3.1 Long-term care - Inpatient	3.2 Providers of ambulatory care - Dental practices	3
2.2 Rehabilitative care - Day	N.E.C.	3
2.2 Rehabilitative care - Day	5. Retailers and other providers of medical goods	3
3.2 Long-term care - Day	N.E.C.	3
2.nec Rehabilitative care - NEC	1.3 Hospitals - Specialized	3
7. Governance & admin	1.nec Hospitals - Other	3
2.4 Rehabilitative care - Home-based	7. Health care system admin.	3
1. nec Curative care - NEC	3.4 Providers of ambulatory care - Ambulatory health care centers	3
6.6 Prevention care - Preparing for disaster and emergency response programs	2. Resid. long-term care fac.	3

1.3.2 Curative care - Outpatient - Dental	1.nec Hospitals - Other	2
3.nec Long-term care - NEC	9. Rest of world	2
3.1 Long-term care - Inpatient	6. Providers of preventative care	2
1.3.3 Curative care - Outpatient - Specialized	1.nec Hospitals - Other	2
1.3.2 Curative care - Outpatient - Dental	3.nec Providers of ambulatory care - NEC	2
1.3.3 Curative care - Outpatient - Specialized	3.nec Providers of ambulatory care - NEC	2
1.2 Curative care - Day	1.nec Hospitals - Other	2
2.2 Rehabilitative care - Day	9. Rest of world	2
7. Governance & admin	3.nec Providers of ambulatory care - NEC	2
2.nec Rehabilitative care - NEC	6. Providers of preventative care	2
3.3 Long-term care - Outpatient	9. Rest of world	2
6.2 Prevention care - Immunization programs	3.2 Providers of ambulatory care - Dental practices	2
6.1 Prevention care - IEC programs	1.nec Hospitals - Other	2
3. Long-term care	3.nec Providers of ambulatory care - NEC	2
7. Governance & admin	3.3 Providers of ambulatory care - Other practices	2
6.5 Prevention care - Epi surveillance and risk and disease control programs	N.E.C.	2
2.nec Rehabilitative care - NEC	2. Resid. long-term care fac.	2
2.2 Rehabilitative care - Day	6. Providers of preventative care	1
7. Governance & admin	3.2 Providers of ambulatory care - Dental practices	1
6.6 Prevention care - Preparing for disaster and emergency response programs	3.1 Providers of ambulatory care - Medical practices	1
3.nec Long-term care - NEC	3. Providers of ambulatory care	1
1. nec Curative care - NEC	4. Providers of ancillary services	1
3.3 Long-term care - Outpatient	6. Providers of preventative care	1
5.nec Medical goods - NEC	6. Providers of preventative care	1
2. Rehabilitative care	3.nec Providers of ambulatory care - NEC	1
2.3 Rehabilitative care - Outpatient	3.nec Providers of ambulatory care - NEC	1
5.1.1 Medical goods - Prescribed meds	1.nec Hospitals - Other	1

1.2 Curative care - Day	3.nec Providers of ambulatory care - NEC	1
1.3.nec Curative care - Outpatient - NEC	3.nec Providers of ambulatory care - NEC	1
1. nec Curative care - NEC	3.2 Providers of ambulatory care - Dental practices	1
1. nec Curative care - NEC	3.3 Providers of ambulatory care - Other practices	1
2.4 Rehabilitative care - Home-based	9. Rest of world	1
2.4 Rehabilitative care - Home-based	N.E.C.	1
2. Rehabilitative care	1.nec Hospitals - Other	1
3. Long-term care	1.nec Hospitals - Other	1
6.nec Prevention care - NEC	3.nec Providers of ambulatory care - NEC	1
6.5 Prevention care - Epi surveillance and risk and disease control programs	3.5 Providers of home health care services	1
1. nec Curative care - NEC	6. Providers of preventative care	1
2.2 Rehabilitative care - Day	3.2 Providers of ambulatory care - Dental practices	1
3.nec Long-term care - NEC	1.3 Hospitals - Specialized	1
2.nec Rehabilitative care - NEC	N.E.C.	1
5.1.2 Medical goods - Over the counter meds	4. Providers of ancillary services	1
1.4 Curative care - Home-based	7. Health care system admin.	1
5.nec Medical goods - NEC	1. Hospitals	1
5.nec Medical goods - NEC	1.1 Hospitals - General	1
5.nec Medical goods - NEC	3. Providers of ambulatory care	1
5.nec Medical goods - NEC	3.4 Providers of ambulatory care - Ambulatory health care centers	1
6.1 Prevention care - IEC programs	3.nec Providers of ambulatory care - NEC	1
2.nec Rehabilitative care - NEC	7. Health care system admin.	1
5.nec Medical goods - NEC	1.3 Hospitals - Specialized	1
5.nec Medical goods - NEC	3.5 Providers of home health care services	1
2.nec Rehabilitative care - NEC	N.E.C.	1
1.2 Curative care - Day	2. Resid. long-term care fac.	1

1.4 Curative care - Home-based	3.1 Providers of ambulatory care - Medical practices	1
2.2 Rehabilitative care - Day	3.1 Providers of ambulatory care - Medical practices	1
2.2 Rehabilitative care - Day	4. Providers of ancillary services	1
2.2 Rehabilitative care - Day	5. Retailers and other providers of medical goods	1
2.2 Rehabilitative care - Day	7. Health care system admin.	1
2.2 Rehabilitative care - Day	8. Rest of economy	1
2.4 Rehabilitative care - Home-based	2. Resid. long-term care fac.	1
2.4 Rehabilitative care - Home-based	3.1 Providers of ambulatory care - Medical practices	1
2.4 Rehabilitative care - Home-based	4. Providers of ancillary services	1
5.1.2 Medical goods - Over the counter meds	1.3 Hospitals - Specialized	1
5.1.2 Medical goods - Over the counter meds	4. Providers of ancillary services	1
5.nec Medical goods - NEC	1. Hospitals	1
5.nec Medical goods - NEC	3. Providers of ambulatory care	1
5.nec Medical goods - NEC	3.4 Providers of ambulatory care - Ambulatory health care centers	1
6.1 Prevention case - IEC programs	3.nec Providers of ambulatory care - NEC	1
6.3 Prevention case - Early disease detection	3.nec Providers of ambulatory care - NEC	1
6.4 Prevention case - Healthy condition monitoring programs	3.nec Providers of ambulatory care - NEC	1
2.nec Rehabilitative care - NEC	7. Health care system admin.	1
